# Contrasting patterns of alpine biodiversity across mountains and taxa worldwide

**DOI:** 10.1038/s41467-026-75210-6

**Published:** 2026-07-03

**Authors:** Lotta Schultz, Ondřej Mottl, L. Camila Pacheco-Riaño, Eline S. Rentier, Pierre Denelle, Stefan Dullinger, Holger Kreft, Davnah Urbach, Mark Snethlage, Riccardo Testolin, Patrick Weigelt, Matteo Anderle, Óscar J. Arribas, Addisu Asefa, Santiago F. Burneo, Carlos Daniel Cadena, Brindusa Covaci, Mihai Covaci, Kiraz Erciyas-Yavuz, Jesús A. Fernández, Astghik Ghazaryan, Arash Ghoddousi, Pranav Gokhale, Stephan Halloy, Tigran Hayrapetyan, Muhammad Zafar Khan, Igor Khorozyan, Shai Meiri, Michele Menegon, Joanne Monks, Agustina Novillo, Jairo Pérez-Torres, Donald Reid, Uri Roll, Paras Bikram Singh, Anil Soyumert, Peter John Taylor, Oscar Thomas, J. Nicolás Urbina-Cardona, Sylvain Ursenbacher, Wei Xu, Nigel G. Yoccoz, Dongru Zhang, John-Arvid Grytnes, Suzette G. A. Flantua

**Affiliations:** 1 Department of Biological Sciences, University of Bergen, Bergen, Norway; 2 Bjerknes Centre for Climate Research, University of Bergen, Bergen, Norway; 3 Department of Botany, Prague, Charles University Czech Republic; 4 Center for Theoretical Study, Prague, Charles University Czech Republic; 5 Department of Biological and Environmental Sciences, University of Gothenburg, Gothenburg, Sweden; 6 Gothenburg Global Biodiversity Centre, Gothenburg, Sweden; 7 Biodiversity, Macroecology & Biogeography, University of Göttingen, Göttingen, Germany; 8 Department of Botany and Biodiversity Research, University of Vienna, Vienna, Austria; 9 Centre of Biodiversity and Sustainability, University of Göttingen, Göttingen, Germany; 10 Global Mountain Biodiversity Assessment, Institute of Plant Sciences, University of Bern, Bern, Switzerland; 11 Centre Interdisciplinaire de Recherche sur la Montagne, University of Lausanne, Lausanne, Switzerland; 12 BIOME Lab, Department of Biological, Geological and Environmental Sciences, Alma Mater Studiorum - University of Bologna, Bologna, Italy; 13 Department of Environmental Science, Radboud Institute for Biological and Environmental Sciences (RIBES), Nijmegen, Radboud University Netherlands; 14 Institute for Alpine Environment, Eurac Research, Bolzano, Italy; 15 IES Castilla, Junta de Castilla y León, Soria, Spain; 16 Ethiopian Wildlife Conservation Authority, Addis Ababa, Ethiopia; 17 Museum of Zoology, Centro de Investigaciones para la Biodiversidad, Pontificia Universidad Católica del Ecuador, Quito, Ecuador; 18 Departamento de Ciencias Biológicas, Universidad de los Andes, Bogotá, Colombia; 19 Centre for Mountain Economy, CBM International University, Cherokee Village, USA; 20 Wildlife Research Institute & Ornithological Research Center, Ondokuz Mayıs University, Samsun, Turkey; 21 Facultad de Zootecnia y Ecología, Universidad Autónoma de Chihuahua, Chihuahua, México; 22 Research Institute of Biology, Yerevan State University, Yerevan, Armenia; 23 Wildlife Ecology and Conservation Group, Wageningen University and Research, Wageningen, the Netherlands; 24 Conservation Biogeography, Humboldt-University Berlin, Berlin, Germany; 25 School of Biological Sciences, Georgia Institute of Technology, Atlanta, USA; 26 New Zealand Ministry for Primary Industries, Wellington, New Zealand; 27 Department of Forestry, Range & Wildlife Management, Karakoram International University, Gilgit, Pakistan; 28 Scientific Center of Zoology and Hydroecology, Yerevan, Armenia; 29 Independent consultant in mammal research and biodiversity conservation, Göttingen, Germany; 30 School of Zoology, Faculty of Life Sciences, Tel-Aviv University, Tel Aviv, Israel; 31 MUSE, Museo delle Scienze, Viale del Lavoro e delle Scienza, 3 Trento Italy; 32 PAMS Foundation, Arusha, Tanzania; 33 Department of Zoology, University of Otago, Dunedin, New Zealand; 34 Instituto de Biodiversidad Neotropical, CONICET-UNT, Cúpulas de Horco Molle, 4107 Tucuman, Argentina; 35 Department of Biology. Faculty of Sciences. Unidad de Ecología y Sistemática (UNESIS), Pontificia Universidad Javeriana, Bogotá, Colombia; 36 Wildlife Conservation Society Canada, Y1A0E9, Whitehorse, Canada; 37 Mitrani Department of Desert Ecology, Ben-Gurion University of the Negev, Midreshet Ben-Gurion, Israel; 38 Biodiversity Conservation Society (BIOCOS Nepal), Laliptur, Nepal; 39 Game and Wildlife Programme, Kastamonu University, Kastamonu, Turkey; 40 Department of Zoology and Entomology and Afromontane Research Unit, University of the Free State, Phuthaditjhaba, South Africa; 41 Pontificia Universidad Javeriana, Facultad de Estudios Ambientales y Rurales. Carrera 7N 40 – 62, Bogotá, Colombia; 42 info fauna, Neuchâtel, Switzerland; 43 University of Neuchâtel, Neuchâtel, Switzerland; 44 Balaton Limnological Research Institute, Tihany, Hungary; 45 DIADE, Université Montpellier, CIRAD, IRD, Montpellier, France; 46 Arctic and Marine Biology, UiT Arctic University of Norway, Tromsø, Norway; 47 School of Biological Science and Technology, Liupanshui Normal University, Guizhou, China; 48 Faculty of Environmental Sciences and Natural Resource Management, Norwegian University of Life Sciences, Ås, Norway

**Keywords:** Biogeography, Ecosystem ecology, Biodiversity

## Abstract

The alpine biome, located at higher elevations of mountains worldwide, supports unique biodiversity and provides important ecosystem contributions to people. Despite the growing recognition of mountain biodiversity in international policy frameworks, substantial gaps remain in our understanding of how alpine biodiversity varies across mountain systems, undermining estimates of its conservation value and consequently effective conservation strategies. Here, we curate a dataset on alpine biodiversity, incorporating expert-validated data on species’ elevational ranges for vascular plants, mammals, birds, and reptiles across 32 mountain ranges worldwide. We show that alpine biodiversity hotspots are concentrated in Neotropical regions, while most temperate regions represent coldspots with lower species richness. These patterns persist whether considering species with broad elevational ranges or only those strictly confined to the alpine zone. Unlike the classical latitudinal gradient of biodiversity, alpine richness patterns show no consistent relationship with latitude, highlighting the importance of regional history, landscape structure, and biogeographical processes.

## Abstract (web summary)

This study compares alpine biodiversity patterns for plants and vertebrates across mountains worldwide using an expert-validated dataset. Authors found strong variation among mountains and taxonomic groups, likely driven by biogeographic history.

## Introduction

Mountains support disproportionately high species richness compared to lowland areas, with tropical mountains standing out as biodiversity hotspots^1–3^. Above the climatic forest line, the alpine biome harbours a distinct biota that holds an important share of global biodiversity^4,5^. These high-elevation ecosystems host numerous endemic species^6,7^ and provide important contributions to people living in and downstream of mountain regions around the world^8,9^. However, global change is rapidly reshaping the alpine biome and its biodiversity^10,11^. Rising temperatures reduce suitable habitat for cold-adapted alpine species and increase competition with species shifting up from lower elevations^12,13^. Simultaneously, intensified anthropogenic activities, such as mining^14^ and pollution^15^, increase the pressure on high-elevation ecosystems worldwide. As a result, the alpine biome is expected to decrease in extent in the coming decades^16^, placing its biodiversity and ecological functions at high risk. Global policy frameworks, including the International Panel of Climate Change (IPCC, AR6 CCP5)^17^, the Intergovernmental Platform on Biodiversity and Ecosystem Services (IPBES)^18^, and the Kunming-Montreal Global Biodiversity Framework of the United Nations (KMGBF)^19^, highlight the urgency of protecting high-elevation ecosystems and their biodiversity. However, formulating evidence-based action plans remains constrained by persistent gaps in biodiversity data and by the fragmentation and limited accessibility of existing information^17^.

Understanding how alpine biodiversity is distributed across and within mountain regions remains an unresolved challenge in global mountain science, with important bearings for conservation efforts. This is the case in large part because cross-mountain comparisons of alpine biodiversity are limited by incompatible data sources, where differences in spatial resolution and format, ranging from range maps to plot data, undermine synthesis efforts. In addition, many datasets lack detailed information on species’ elevational ranges. This information is crucial for identifying which species are true alpine specialists (i.e., restricted to alpine ecosystems) and which also occupy a wider range of elevations, including areas below the alpine zone^9,20^. Also, past attempts to study alpine diversity patterns globally focus disproportionately on plants^21,22^ and birds^23,24^, leaving other taxonomic groups, such as mammals and reptiles, underrepresented. Moreover, research efforts are unevenly distributed across mountain systems, with many well studied regions located in temperate regions, despite tropical mountains hosting substantially higher biodiversity^25^. While previous studies have linked alpine biodiversity to environmental factors^26^, diversification dynamics^27^, and species traits^23^, a cohesive biogeographic perspective across regions and taxa is still lacking. Advancing a global understanding of alpine biodiversity demands systematic, cross-taxon datasets on species distributions and elevational ranges that enable direct comparisons across the world’s mountain ranges.

**Fig. 1:**
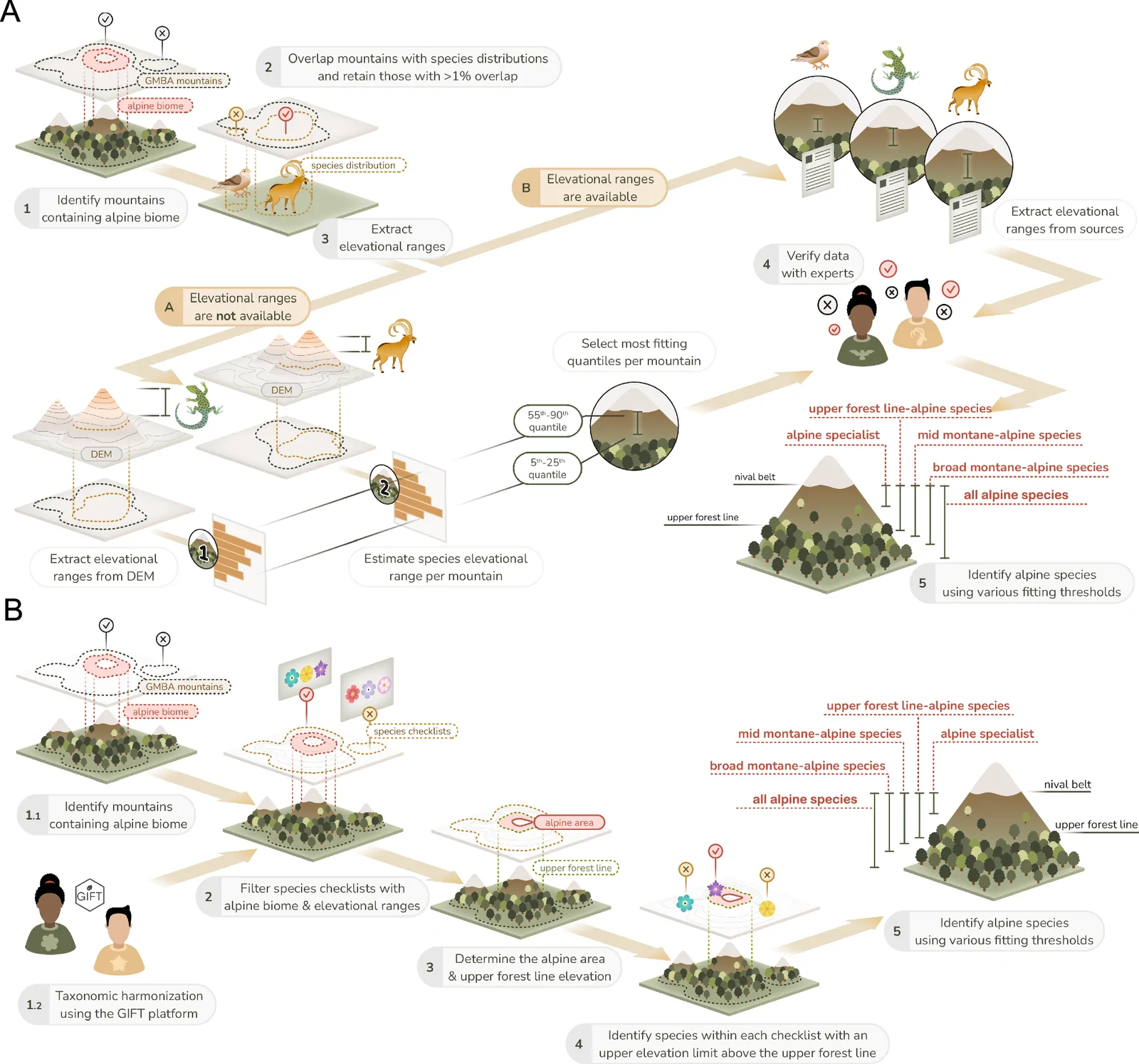
Workflow for identifying occurrences and elevational ranges for vertebrates and vascular plants within mountain ranges. **A** For mammals, birds, and reptiles, we used GMBA-delineated global mountain ranges^28^ containing alpine biome areas (step 1) and overlaid them with range polygons (step 2). Elevational ranges were extracted from literature sources at the mountain range level when available (step 3B); otherwise, they were estimated using a Digital Elevation Model (DEM) (step 3 A)^52^. For the latter, the ranges of species unique to a single mountain range were spatially intersected with mountain polygons, and elevation data were extracted from these intersections (step 3.1), and compared with verified elevation data where available (step 3.2). Quantiles representing the species’ lower and upper elevational limits were selected (step 3.3) to estimate these limits within each mountain range for which elevational ranges were unavailable in literature sources. Species occurrences and their elevational ranges were then validated by regional experts (step 4). Finally, species were grouped within each mountain range into montane-alpine categories based on elevational ranges (step 5). **B** For plants, we derived regional checklists from the GIFT database^29,69^ that (i) overlapped with our alpine biome (step 1.1), (ii) had been taxonomically standardised (step 1.2), and (iii) included regional elevational ranges per species (step 2). We determined the alpine area and elevation of the UFL within each checklist (step 3) and selected species whose highest elevation exceeded the regional UFL (step 4). Those species were then grouped into categories based on their lower and upper elevational limits relative to the UFL. The categories included all alpine species, broad montane-alpine species, mid-montane-alpine species, UFL-alpine species and alpine specialists (step 5). Figure courtesy of Miranta Kouvari of Science Graphic Design.

Our study addresses this need by: (1) developing an expert-validated dataset of multi-taxon alpine biodiversity across mountain ranges globally, and (2) using this dataset to uncover patterns in the distribution and elevational range preferences of vertebrates (mammals, birds, and reptiles) and vascular plants (hereafter plants). The foundation of our study is an open-access checklist of alpine biodiversity that integrates species lists and elevational information at the mountain range level (sensu Snethlage et al. 2022^28^). For vertebrates, we use spatial analyses and data gathered from the literature to compile species lists and elevational ranges, reviewed by 32 regional experts worldwide (Fig. 1A). For plants, we derive 80 spatially delineated checklists from mountain ranges containing an alpine zone from the Global Inventory of Floras and Traits (GIFT)^29^ (Fig. 1B), which integrates quality-checked and taxonomically harmonised regional plant checklists (Supplementary Data 2).

Alpine species that occur across broader elevational gradients may be less sensitive to shifting conditions, while alpine specialists can be particularly vulnerable to habitat loss and fragmentation, given their dependence on the increasingly limited alpine biome^30^. We classify all vertebrate and plant species into categories of alpine specialisation according to how confined their elevational ranges are to the alpine zone, using mountain-specific elevational cut-offs corresponding to temperature thresholds relative to the upper forest line (UFL; see “Methods” for details). The five categories are (i) all alpine species (species with upper elevational limit above UFL, no restriction based on their lower limit), (ii) broad montane-alpine species (upper elevational limit above UFL, lower limit above elevations corresponding to 6 °C below UFL), (iii) mid montane-alpine species (upper elevational limit above UFL, lower limit above elevations corresponding to 4 °C below UFL), iv) UFL-alpine species (upper elevational limit above UFL, lower limit above elevations corresponding to 2 °C below UFL), and v) alpine specialists (species fully restricted to the alpine zone). In the main text we focus on all alpine species and alpine specialists (hereafter referred to as specialists), with findings for other categories available in the Supplementary Information (Supplementary Figs. 3–6 and Supplementary Data 3, 4).

Using these categories, we estimate and compare alpine species richness across 32 mountain ranges worldwide (See Methods, Supplementary Fig. 1 and Supplementary Data 1, 2). Recognising that the extent of alpine areas varies significantly across mountains, and that area strongly influences richness^20,31^, we complement absolute richness with an area-corrected measure of relative richness derived from a Species-Area-Relationship (SAR) model. To explore latitudinal trends in both richness measures, we fit Generalised Additive Models (GAMs) using the latitudinal centroid and latitudinal extent of each alpine area as predictors. Throughout this study, we use the terms hotspots and coldspots to describe mountain ranges with the highest and lowest richness levels in our analyses, respectively. These terms refer solely to richness within our dataset and do not imply differences in conservation importance, as regions with lower overall richness may still be of great global and regional importance. In addition, we classify latitudes as low (0°–30°), mid (30°–60°), and high (above 60°) in both the Northern and Southern Hemispheres.

Our study reveals the global heterogeneity of alpine biodiversity within and among mountain ranges, providing a stronger foundation for targeted conservation in alpine ecosystems^32^, and opening new opportunities for research on alpine biogeography, and the impacts of past and future climate change on high-elevation biodiversity. We integrate mountain-specific elevational data and expert knowledge to refine coarse information on species distributions and to address geographical bias in data quantity and reliability. Although data gaps remain, this approach improves insights into species’ elevational preferences and enables analyses of alpine biodiversity patterns across mountain systems.

## Results

### Alpine species numbers

Of the 27,803 known vertebrate species in the world, we find that 2165 species (7.8%) occur in the alpine biome that covers approximately 3% of the Earth’s total terrestrial surface^33^. Of these 1173 are bird species (constituting 10.4% of the 11,195 bird species globally), 711 species are mammals (10.8% of 6544 species globally), and 281 reptile species (2.8% of 10,064 species globally). Among the species found in the alpine biome, 360 species are alpine specialists. Of these specialists, 147 species are birds (12.5% of all bird species found in the alpine biome), 93 species are mammals (13.1%), and 120 species are reptiles (42.8%). Underscoring the importance of the alpine biome for global biodiversity, we further find that 91% of the alpine specialist species of vertebrates are confined to a single mountain range, with birds showing the highest proportions of species restricted to a single range (Supplementary Fig. 3A). Including all alpine species reveals a tendency towards more species with broader geographic ranges. However, 65% are still confined to a single mountain range (Supplementary Fig. 3A). Some of these species may also be present in mountain ranges without an alpine zone that are not included in our analyses. Disproportionately high concentrations of geographically restricted species in alpine zones were also found in previous research^6,7^, aligning with our finding that many alpine species are confined to a small number of mountain ranges.

We identify 14,649 plant species occurring in the alpine biome, representing c. 4% of all known vascular plants worldwide^34^. As the GIFT checklists do not cover every alpine area globally, this number cannot be directly compared to our total estimates for vertebrates. Of the plant species found in the alpine biome, 17% (2554 species) are further classified as alpine specialists. Similar to vertebrates, a large proportion (70%) of alpine specialists are geographically restricted and are found in only a single GIFT region (Supplementary Fig. 3B). By contrast, 53% of the full alpine plant richness are confined to a single region.

### Global hotspots and coldspots of alpine biodiversity

**Fig. 2:**
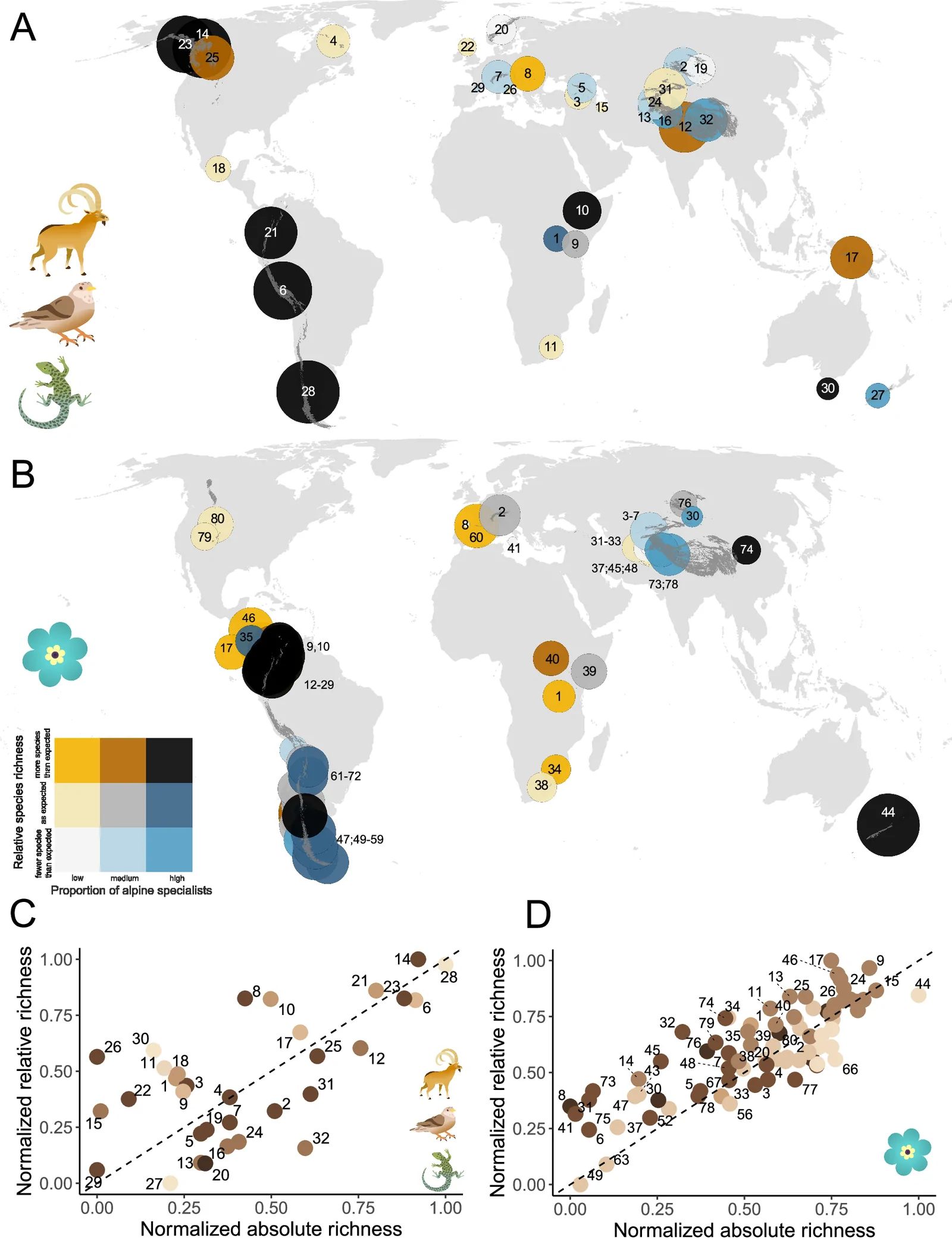
Global distribution of vertebrate and vascular plant richness (absolute and relative) across mountain ranges worldwide and proportions of alpine specialists. Bubble size represents the absolute richness of all species for individual mountain ranges (**A** vertebrates, **B** vascular plants). Bubble colour reflects the proportion of alpine specialists (i.e., species restricted to the alpine biome) along the X-axis, and relative richness (i.e., richness relative to alpine area) along the Y-axis, calculated as residuals from the species-area relationship. The darkest bubbles highlight alpine hotspots, where both the proportion of alpine specialists and relative richness are the highest. The lighter bubbles indicate areas with lower proportions of specialists and fewer species than expected based on their alpine area. Colour bins are defined by quartiles. The proportion of alpine specialists’ ranges between 0–38% on a logarithmic scale, with values increasing from low to high across the X-axis of the legend. Colours indicate whether species richness is less than expected (negative residuals), as expected (around zero residuals), or higher than expected (positive residuals). Normalised absolute and relative richness for vertebrates (**C**) and plants (**D**). Absolute and relative richness are scaled between 0 and 1 based on their minimum and maximum values (i.e., (richness – min(richness)) / (max(richness) – min(richness))). Points in the top right along the diagonal line depict mountain ranges with the highest relative and absolute richness. Points below the diagonal indicate mountain ranges where absolute richness is greater than relative richness, while points above the diagonal indicate the opposite. The colour gradient represents latitude, ranging from light grey (lowest latitude) to black (highest latitude). Numbers correspond to mountain ranges for vertebrates and GIFT regions for plants, as detailed in Supplementary Data 1 and 2, respectively.

We find that global alpine biodiversity varies widely across and within mountain ranges, with distinct regional hotspots and coldspots for vertebrates and plants (Fig. 2). Yet, the overall global distribution of these hotspots and coldspots remains relatively consistent across the five alpine specialisation categories we defined (Fig. 2 and Supplementary Figs. 4–6). The Neotropical alpine ecosystems in the Andes consistently emerge as a hotspot, showing exceptionally high absolute and relative richness across all taxonomic groups (vertebrates and plants) (Fig. 2C, #6,21,26; Fig. 2D #9,12-29; the # symbol refers to the mountain range ID in the figures and Supplementary Data 1–4). Neotropical alpine ecosystems also have the highest proportions of alpine specialists in both vertebrates and plants (Fig. 2A, B). In contrast, several alpine areas at higher latitudes, such as the European Alps and the North European Highlands are alpine biodiversity coldspots, harbouring fewer species than expected based on their area. Here, alpine species richness is largely driven by species with broad elevational ranges that extend below the UFL, while relatively few species are restricted to the alpine zone (Fig. 2 and Supplementary Figs. 4–6). At similar latitudes, the Central Asian alpine ecosystems exhibit high absolute species richness, particularly on the Tibetan Plateau and the Himalaya, and a relatively high proportion of alpine specialists (Fig. 2A, #32, 12). Although these areas are proposed as temperate biodiversity hotspots^21^, we show that when accounting for their vast alpine extent, they host lower-than-expected richness.

### Complex patterns across mountains and taxonomic groups

Our findings also demonstrate that alpine biodiversity patterns are not uniform across taxonomic groups. In East Africa, for example, the Ethiopian Highlands show high relative richness for mammals and birds, despite having limited and fragmented alpine areas, while plants and reptiles show much lower richness (Fig. 2B, D and Supplementary Fig. 7). By contrast, the isolated volcanic mountains in Kenya and Uganda support high relative plant richness with notable proportions of alpine plant specialists (Fig. 2B, D #1, 40), but only moderate richness for mammals and birds and low relative richness for reptiles (Fig. 2A). Other Afroalpine ecosystems, such as the Drakensberg and the Albertine Rift, emerge as relative richness coldspots for reptiles (Supplementary Fig. 7 #1, 11), while still supporting moderate relative richness for plants and comparatively high proportions of alpine specialists among mammals.

Similar taxon-specific variation in alpine biodiversity is also apparent in mountain ranges across Northern latitudes. Along the West Coast of North America, for example, alpine ecosystems exhibit only moderate relative plant richness but high absolute and relative vertebrate richness, with particularly high numbers of alpine specialists among vertebrate groups (Fig. 2A, C, #23, #14). In contrast, the Northeastern alpine ecosystems of North America show high relative richness for mammals but support fewer alpine specialists, while bird richness is low and reptiles are absent from this alpine zone (Supplementary Fig. 7 #4). At similar latitudes, the Central Asian alpine ecosystems, such as the Tibetan Plateau and the Hindu Kush highlands of Afghanistan, show high absolute but low relative richness in birds, mammals (Fig. 2C) and plants (Fig. 2D, #31-32,37), whereas reptile absolute richness is generally lower in these regions. New Zealand presents a unique pattern: while it is a hotspot for alpine plant diversity (Fig. 2B, D #44), patterns vary across vertebrate groups (Supplementary Fig. 7). Here, reptile richness and specialisation are comparatively high, birds exhibit intermediate species richness, and alpine mammals are entirely absent. This absence, however, reflects New Zealand’s overall mammalian fauna, which consists only of three bat species and no other native terrestrial mammals^35^.

### Latitudinal trends of richness - Does climatic similarity decouple the alpine biome from global biodiversity gradients?

Although alpine biodiversity hotspots and coldspots are scattered across the globe, alpine ecosystems themselves share similar climatic characteristics, including low temperatures and associated constraints on vegetation growth^9^. Because global biodiversity patterns are often structured by latitude^36^, the alpine biome provides a natural test whether the latitudinal gradient persists under relatively uniform climatic conditions. If climate alone would drive latitudinal patterns of biodiversity, the relationship between species richness and latitude should weaken. If, however, other latitude-linked factors, such as differences in diurnal and seasonal variations in temperature^37,38^, also shape biodiversity, a latitudinal gradient in alpine species richness may still emerge.

**Fig. 3:**
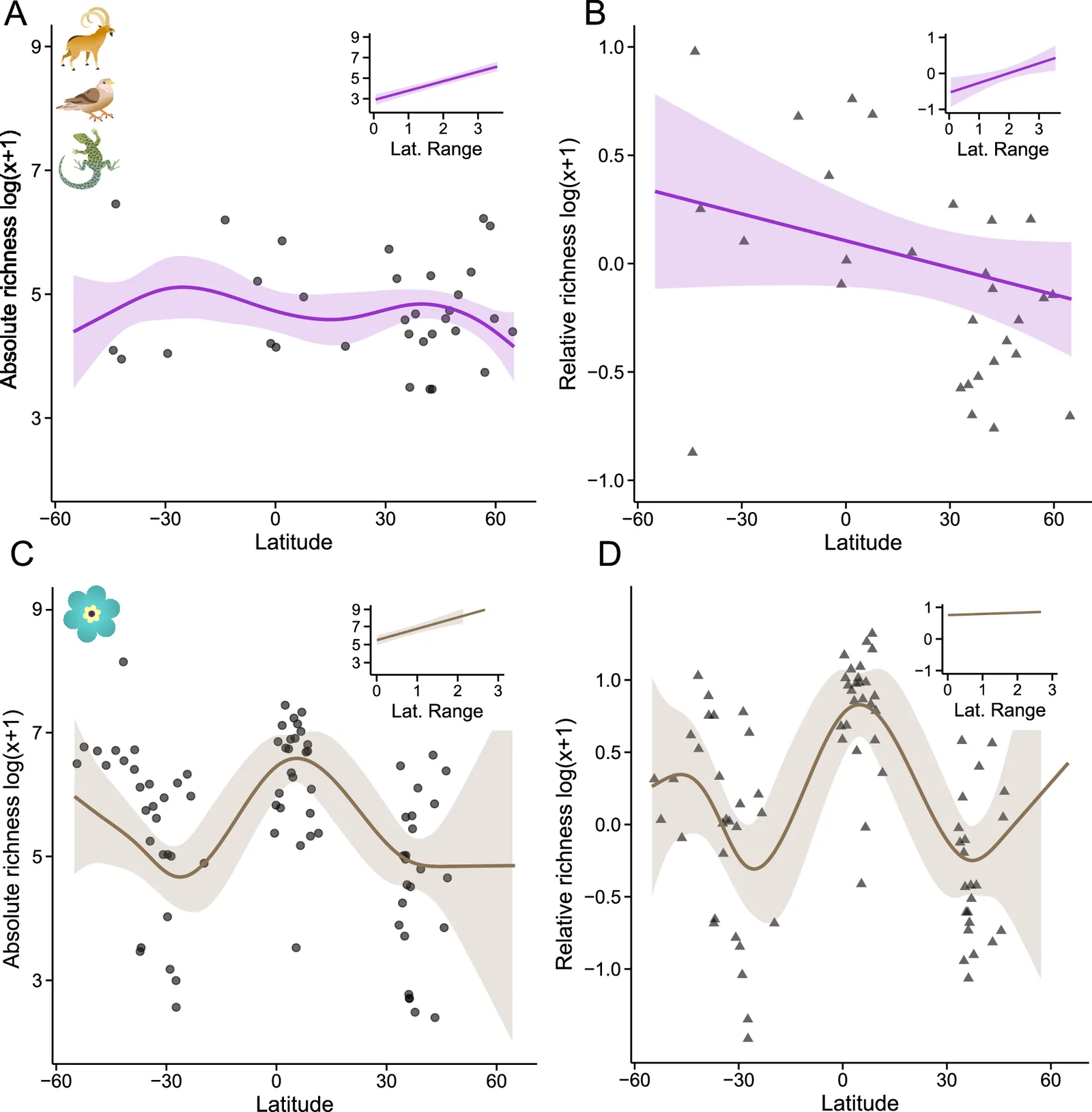
Latitudinal trends of alpine biodiversity. Vertebrates (**A**, **B**) and vascular plants (**C**, **D**) are shown as absolute richness (**A**, **C**) and relative richness (**B**, **D**). Points in the absolute richness plot and triangles in the relative richness plot indicate the latitudinal centroids of alpine areas within mountain ranges. The top-right inset in each plot shows the effect of latitudinal range (i.e., the distance between the southernmost and northernmost points of each mountain range). The Y-axis represents the log1p-transformed absolute richness (**A**, **C**) or relative richness adjusted for area (**B**, **D**). Lines indicate GAM fitted to latitude and richness, with shaded areas representing 95% confidence intervals.

We find that alpine species richness follows no clear latitudinal gradient with hotspots and coldspots occurring across latitudes (Fig. 3). For vertebrates, absolute richness shows a clear nonlinear relationship with latitude, with a peak around 25 °S and a secondary peak near 40 °N. Absolute richness also increases with latitudinal range of the alpine area (i.e., the difference in latitude between its northernmost and southernmost boundaries), reflecting higher species counts in alpine areas that span broader north-south gradients (overall model explained 81.3% deviance; latitude smooth term: F(4.33,5.31) = 1.26, *p* = 0.31; latitudinal range effect: *β* = 0.92, t(30) = 9.88, *p* < 0.001; Fig. 3A). However, when richness is scaled by alpine area, these patterns change. Relative richness declines linearly with latitude, and the effect of latitudinal range disappears (overall model explained 25.4% deviance; latitude smooth term: F(1,1) = 2.45, *p* = 0.13; latitudinal range effect: β = 0.27, t(30) = 2.77, *p* = 0.01; Fig. 3B). This suggests that differences in alpine area size explain much of the apparent latitudinal pattern.

Plant richness shows more consistent latitudinal patterns between absolute and relative metrics (Fig. 3C, D). In both cases, richness peaks near the tropics and declines at intermediate latitudes (e.g., 25°N and 25°S). As for vertebrates, the effect of latitudinal range is evident for absolute richness but disappears once richness is scaled by area (absolute richness: overall model explained 55.6% deviance; latitude smooth term: F(4.80,5.75) = 9.30, *p* < 0.001; latitudinal range effect: *β* = 1.26, t(78) = 5.68, *p* < 0.001; relative richness: overall model explained 46.9% deviance; latitude smooth term: F(5.41,6.40) = 9.44, *p* < 0.001; latitudinal range effect: *β* = 0.04, t(78) = 0.29, *p* = 0.77).

### Perspectives on global alpine biodiversity

Our results reveal global variation in patterns of alpine biodiversity, characterised by distinct hotspots and coldspots, the absence of a consistent latitudinal pattern, and differences in alpine specialisation among taxonomic groups. This raises key questions about the processes that shape alpine biodiversity. Many of these processes are likely linked to differences in geological history^22^, mountain topography^16^ and the legacy of past climate dynamics^39^. Repeated glacial advances and retreats reshaped alpine ecosystems through ice cover and erosion, altering their degree of connectivity and isolation over the last millions of years^39,40^. These dynamics interacted with mountain shape and topography to determine whether glacial and interglacial periods fragmented alpine ecosystems into isolated refugia or, conversely, expanded and connected alpine areas^16,39,40^. Such processes influenced species persistence, migration, hybridisation, extinction, and speciation over evolutionary timespans, ultimately shaping patterns in alpine biodiversity^41^.

Yet, important differences in these biogeographic processes exist among mountains. During glacial periods, extensive glaciers in the European Alps restricted alpine ecosystems to peripheral refugia around the border of the icesheet^42^. In contrast, alpine ecosystem connectivity increased in the Northern Andes^43^ and African mountains^44^ during these periods, although to varying degrees^45^ shaped by mountain-specific topography^16^. Mountain ranges with limited topographic relief, such as the Drakensberg, likely offered fewer stable refugia and reduced habitat heterogeneity, restricting the persistence of alpine communities through past climatic fluctuations^44^, which may explain the lower absolute species richness observed in this region. In contrast, geologically young and topographically complex mountain ranges such as the Northern Andes, with their many peaks, ridges and high-elevation plateaus, created dynamic mosaics of high connectivity and fragmentation (sensu flickering connectivity system^41^), providing refugia and cradles for species to persist and speciate^1,3,43^. These processes likely underlie the exceptionally high alpine biodiversity observed in these regions. A comparable pattern is evident in North America, where the western Cordillera had non-glaciated refugia during the Pleistocene, allowing species to persist and diversify, while the fully ice-covered eastern ranges lacked such refugia, leading to lower present-day alpine species richness with less alpine specialists^46^.

Despite these hypothesised relationships between mountain biogeographic history and alpine biodiversity, the extent to which regional differences in mountain uplift and climate-driven range dynamics have shaped present-day alpine biodiversity (Fig. 2) remains largely unexplored in a quantitative framework.

### Data limitations

Our dataset lays the groundwork for future studies on alpine biogeography and future biodiversity assessment across the globe. However, like most large-scale biodiversity compilations, it holds certain limitations and biases. One is that the range maps we used rely on sampling efforts, which remain uneven across the globe and are often concentrated in regions with more established research infrastructure (e.g., USA, Australia, and Europe)^47^. Range maps are therefore more accurate in some regions than in others. In addition, the spatial resolution of these maps does not allow precise approximations of where species actually occur within individual mountain ranges or across elevational gradients.

To address these limitations, we incorporated elevational ranges as an additional dimension of species distributions, allowing us to identify which species are likely to be found in the alpine zone of each mountain range. A key strength of this approach lies in the involvement of regional experts who validated the regional elevational distribution and occurrence of species. At the time of analysis, 57% of the species lists were reviewed, reflecting the availability of experts across regions at the time of analysis (Supplementary Fig. 2). Nevertheless, the absence of validation in some regions may have left some inaccuracies in species elevational ranges and occurrences. For example, the comparatively high reptile richness observed in western North American mountain ranges, contrasted with the apparent absence of reptiles in eastern North American mountain ranges, is likely an artefact, stemming from imprecise range maps and the lack of validation. Similarly, the global coverage for plants remains incomplete, with certain regions, such as Central Asia, still underrepresented (Supplementary Data 2), and with additional plant species likely remaining to be discovered, particularly in tropical mountain regions.

For birds, we used resident ranges rather than breeding ranges, a necessary compromise due to the limited availability of high-resolution seasonal data. While this approach ensures broader global coverage and aligns with our aim to capture species that occupy alpine zones year-round, it excludes migrants that breed in alpine ecosystems but overwinter elsewhere, particularly in temperate regions.

### Future directions

Our study presents a global synthesis of multi-taxon richness patterns of the alpine biome, providing new insights into the distribution and diversity of alpine vertebrates and plants. By integrating data across regions and taxa, we uncover a previously unrecognised global mosaic of alpine biodiversity, revealing distinct hotspots and coldspots of high-elevation biodiversity across taxonomic groups.

Our findings underscore the need to disentangle the regional dynamics of individual mountain ranges to understand how alpine biotas are shaped and reshaped by environmental change. Future research must move beyond broad approaches to probe the ecological processes underpinning alpine diversity, including how diversification, dispersal barriers, and biogeographic legacies interact to define species pools, elevational limits, and their sensitivity to environmental changes.

Our open-access dataset provides a foundation for advancing such future research. By providing comparable data across mountain systems worldwide, we aim to establish a consistent global baseline that can continue to be updated and spatially assessed in relation to climate projections, land-use scenarios and species’ threat assessments along elevational gradients. Our work enables the identification of alpine ecosystems where biodiversity is concentrated, underrepresented or potentially at risk, to ultimately support the development of region-specific conservation priorities and management actions.

Finally, we see this work as a catalyst for continued collaboration with regional experts to improve data coverage, refine elevational ranges, and expand taxonomic representation and accuracy. Only by connecting regional expertise worldwide, we can build the evidence base needed to inform global policy frameworks, such as the IPCC, IPBES, and the Kunming–Montreal Global Biodiversity Framework, and ultimately safeguard high-elevation ecosystems and their contributions to future generations.

## Methods

All analyses described in the following sections were conducted using R (version 4.4.2)^48^, and the complete workflow for data processing and creating our global alpine species dataset is openly available as a Zenodo release on GitHub (https://zenodo.org/records/14925032). The complete checklist of alpine species compiled for this study is available through the GMBA Mountain Portal (https://mountainbiodiversity.org/) and on Zenodo (https://zenodo.org/records/20183570).

### The alpine biome across mountains worldwide (Fig. 1, step 1)

To define global mountain ranges, we used the latest delineation provided by the Global Mountain Biodiversity Assessment (GMBA)^28^. This inventory provides a hierarchical categorisation that aggregates mountain ranges from local to global scales. Specifically, we selected GMBA v.2.0 Level 03 for its detailed spatial resolution and suitability in delineating global species distributions in mountains. To spatially delineate the alpine biome globally, we followed the climate-delimitated approach by Kandziora et al. (2024)^49^ based on the high-resolution Köppen-Geiger climate maps^50^. Specifically, we extracted Köppen-Geiger Class 29 (ET; Polar, Tundra) for each GMBA mountain range to represent the alpine biome. This class corresponds to areas where the mean temperature of the warmest month is below 10 °C, i.e., conditions that physiologically limit tree growth. In this context, we refer to the outer boundary of these polygons as the upper forest line (UFL), rather than the treeline. The latter includes the uppermost tree-size individuals, whose growth is strongly influenced by local site conditions^5^. Modelling and predicting its precise location can therefore be challenging due to patchiness and limited spatial resolution in global climate datasets. In contrast, using the upper limit of the continuous montane forest provides a clearer ecological boundary between forest and alpine ecosystems and a more consistent representation at the scale of our climate and distribution data, making it more appropriate for our approach.

We then calculated the mean annual temperature based on CHELSA (version 2.1)^51^ and elevation (Digital Elevation Model, DEM, GMTED10)^52^ at which the present-day UFL (i.e., climatological mean for 1991-2020)^50^ is located. In total, we selected 32 mountain ranges containing an alpine area (Supplementary Fig. 1 and Supplementary Data 1). We excluded arctic and subarctic regions because they form a separate zonobiome^53^ that is shaped by latitude rather than elevation^4^. Furthermore, we recognise that a clearly defined UFL might be absent in some mountain regions due to local environmental factors such as frequent fires and steep topography, as for instance seen in the Drakensberg Mountains of South Africa^54^. In these cases, the UFL we reference here represents a potential or climate-delineated boundary rather than a naturally occurring one. In addition, although in this study we refer to all ecosystems above the UFL as alpine, they are known by different regional names. In the tropics, for instance, they include the páramo and puna in the Andes and the Afro-alpine zone in Africa^55^. For clarity, however, we here use a unified terminology throughout.

### Collection of mammal, bird, and reptile data

#### Distribution data (Fig. 1A step 2)

For mammals and reptiles, we gathered global native distribution ranges for all extant species from the Mammal Diversity Database (MDD, version 1.11; *n *= 6544 spp.)^56^, and the Global Assessment of Reptile Distribution (GARD, version 1.7; *n* = 10,064 spp.)^57,58^, respectively. For all extant birds, we used the BirdLife International native resident distribution ranges (version 2022.2) defined as the area where species are known or thought to be resident throughout the year or visit regularly during the breeding season (*n* = 11,195 spp.)^59^. Due to limited data availability, we focused on resident distribution ranges as defined by BirdLife International, which represent the year-round geographic ranges of non-migratory species and include their breeding areas^59^. Long-distance and local breeding migrants that disperse seasonally were excluded because our aim was to include only those species that use the alpine zones as their primary habitat throughout the year. A valuable complementary reference to our data is the list of alpine breeding birds available from DeZwaan et al. (2020), which focuses exclusively on breeding occurrences^23^.

Mammal taxonomy followed the MDD, which is based on Mammal Species of the World (3^rd^ edition)^60^. Bird taxonomy followed the HBW/BirdLife Taxonomic Checklist of the Birds of the World (V. 9.0)^61^, and reptile taxonomy followed the global species list curated by the Reptile Database^62^.

We overlapped each species’ distribution with the GMBA mountain ranges and included those with at least 1% of their range within a given mountain range (*n* = 16,022 species, 7525 birds, 3952 mammals and 4546 reptiles). We chose this threshold pragmatically to exclude negligible or borderline overlaps while still capturing species that occupy a portion of mountain habitats.

Due to data limitations, amphibians were not included in this version of the dataset. However, we acknowledge that this group serves as a valuable indicator of environmental changes in mountain regions and may be included in future refinements of the dataset.

#### Elevational range data (Fig. 1 step 3)

To obtain regional information of species elevational ranges at the scale of individual mountains, we first compiled elevational range data for our selected set of mountain species using the machine-readable version of the Handbook of Mammals of the World (HMW)^63,64^, Squambase^65^, and the global database of bird elevational ranges^24^. While these sources provide broad geographic coverage, regional elevational data remained unavailable for 47% of the mountain species in our dataset. Moreover, feedback from local experts (see next step) revealed that certain global datasets may not accurately capture regional elevational limits in some areas (e.g., forest-dwelling birds assigned elevations well above the permanent snowline). This emphasised the necessity of expert validation and region-specific information to refine global distribution data and ensure that mountain-specific information is considered where possible.

To fill in the missing information of local elevational ranges (i.e., for species distributed across multiple mountain ranges or for those which data were missing in our primary sources), we adapted the method by Quintero and Jetz (2018)^24^ and determined elevational ranges within each relevant mountain range using a DEM (GMTED2010) at 30 arc seconds resolution:

- Step i: We identified species unique to a single mountain range (e.g., only in the European Alps) with verified elevation data in our sources.
- Step ii: We spatially overlaid (i.e., intersected) these species’ ranges with all mountain ranges containing the alpine biome, after which elevations were extracted from these intersections using the DEM. This step was performed individually for each species for the corresponding mountain range. This allowed us to obtain the elevation values within each species distribution for each corresponding mountain range.
- Steps iii and iv: To estimate the lower and upper elevational limits for each species within each mountain range, we used specific quantiles (in 5^th^ quantile intervals) of these elevation values. By doing so, we avoided simply taking the extreme minimum and maximum values, as it may overestimate or underestimate elevational ranges by capturing the highest and lowest points within the area where a species’ distribution range overlaps a given mountain range. Since the quantile that best represents a species elevation range can vary between mountain ranges, we compared the different quantiles against known elevation data from primary sources. We selected the quantile with the smallest mean deviation from this known data, which ensured that our estimates were tailored to the unique topography of each mountain range. This comparison allowed us to identify the quantile that best matched the known elevation ranges for species within each specific mountain range.
- Step v: This selected quantile was then used to estimate elevation ranges for species in the same mountain range that lacked sufficient data in the literature. This process was repeated for all mountain ranges in our dataset.

#### Data validation by regional experts (Fig. 1 step 4)

Regional experts reviewed and validated vertebrate species lists for each mountain range to improve data quality. Experts were identified through the Global Mountain Biodiversity Assessment (GMBA) and recommendations from others (i.e., “snowballing approach”) and specialised in specific taxonomic groups, mountain ranges, or both. The expert network consisted of 32 specialists representing 17 mountain ranges worldwide (Supplementary Fig. 2). All experts were provided with standardised instructions to refine the dataset. Individually or in collaboration with others, the experts corrected species occurrences and adjusted mountain range-specific elevational extents for the regions where they had expertise.

#### Categorisation of alpine vertebrates (Fig. 1 step 5)

We selected vertebrate species from our checklists whose upper elevational limits extend beyond the UFL in each mountain range. Since many alpine species also inhabit zones below the UFL, we used variable cut-offs for inclusion based on their lower elevational limits. Specifically, we examined how far a species’ lower range limit extends into the life zones below the UFL, upper montane ( ~ 4 °C above the temperature at the UFL) and lower montane ( ~ 8.6 °C above the temperature at the UFL)^9^. We calculated how much elevation change (in metres) is needed for a 1 °C temperature change, using the specific rate of temperature drop with elevation (adiabatic lapse rate) for each mountain range to account for geographic variation in temperature-elevation relationships. For this, we extracted mean annual temperature data for each mountain range from CHELSA (version 2.1)^51^. We then fitted linear models to the temperature-elevation data and used the slope (the coefficient) of these models as the lapse rate for each mountain range.

We then categorised species based on their elevational limits relative to the UFL, assigning the following arbitrary names for simplicity. The categories we assigned are nested, meaning that each more specific group is included within the broader ones above it.

A) All alpine species: Species with an upper elevation limit above the UFL and a lower limit that can extend across the full elevational gradient of a mountain range.
B) Broad Montane-Alpine Species: Species with an upper elevation limit above the UFL and a lower limit extending within 6 °C below the UFL.
C) Mid-Montane-Alpine Species: Species whose upper elevation limit surpasses the UFL, with a lower limit extending within 4 °C below the UFL.
D) UFL-Alpine Species: Species that mainly occur above the UFL, but their lower elevation limit extends within 2 °C below the UFL.
E) Alpine Specialists: Species restricted entirely to zones above the UFL, with both their lower and upper elevation limits falling within the alpine zone.

#### Estimating alpine vertebrate richness in the alpine biome worldwide

To account for the trend that larger alpine areas may support more species^31^, we related the size of the alpine area within each mountain range to the number of species it contains. This relationship, known as the Species-Area Relationship (SAR), is commonly expressed as a linear relationship on a log-log scale: log(*S*) = log(*c*) + *z* log(*A*), where S is species richness, A is alpine area, c is a constant, and z is the slope describing how species richness increases with area^66^. We estimated the alpine area using the geodesic calculation method, which accounts for the Earth’s curvature, and fitted a linear model on the log-transformed data to quantify this relationship. *Z*-values derived from the global slopes of the SAR are: all alpine species = 0.23, alpine specialists = 0.30, UFL-alpine = 0.34, mid-montane-alpine = 0.38, and broad montane alpine = 0.37 (Supplementary Fig. 8A). Finally, we calculated the residuals to quantify the degree of deviation between observed vs predicted species richness. This process was repeated for each previously defined category of alpine species.

In our study, we refer to the residuals calculated from the SAR as relative richness and to observed richness as absolute richness. Although SAR is a widely applied framework in ecological research and conservation applications^67^, the constants are known to vary regionally and by taxon due to factors such as habitat heterogeneity, species dispersal abilities and historical biogeography^68^, and should therefore be interpreted with caution. However, the SAR provides a crucial baseline for our study. Since absolute richness numbers are often influenced by the sheer size of the alpine biome, accounting for how species richness scales relative to the highly variable extent of alpine areas worldwide is essential to accurately describe global richness patterns.

### Collection of plant data

We first gathered regional plant checklists assembled by experts for a previous study^38^ and added them to the Global Inventory of Floras and Traits (GIFT) database^29^. Using the GIFT R package^69^, we then sourced 80 regional checklists of native vascular plants from GIFT version 3.2 for mountains that included the alpine biome and contained regional-specific elevational data (Supplementary Data 3). GIFT checklists are taxonomically standardised using the World Checklist of Vascular Plants (WCVP) taxonomic backbone^34^ and cover various geographic and political units. GIFT is continually updated with new regional checklists. However, at the time of our analysis, plant data was unavailable for some mountain ranges (e.g., in Central Asia) for which vertebrate data was available, leading to occasional mismatches in data coverage between plants and vertebrates in our results. Unlike our vertebrate data, the plant checklists were not revalidated for this study as they had already undergone quality checks before their integration into the GIFT database.

#### Categorisation of alpine plants

For each checklist, we calculated the extent of the alpine area and determined the elevation of the UFL using our delineation of the alpine biome as described above (Fig. 2 step 3 and Supplementary Data 2). We identified alpine plants as species whose upper elevational limits exceed the regional UFL (Fig. 2 step 4). Following the same framework as for vertebrates, we categorised the plants into the same five groups (Fig. 2 step 5).

#### Estimation of alpine plant richness in the global alpine biome

We applied the same approach as for vertebrates, fitting a log-log scaled linear species-area relationship model for each alpine category. The z-values derived for plants on a global scale are: all alpine species = 0.31, alpine specialists = 0.38, UFL-alpine = 0.42, mid-montane-alpine = 0.46, and broad montane alpine = 0.40 (Supplementary Fig. 8B). For each category of alpine plants, we then calculated residuals to determine how observed species richness deviated from the model’s predictions.

### Latitudinal trends of alpine biodiversity

To identify latitudinal trends in absolute and relative richness for vertebrates and plants, we fitted Generalised Additive Models (GAMs) using the mgcv R-package^70^. Latitude was included as the main explanatory variable and modelled as a smooth curve to capture non-linear trends in species absolute and relative richness. Because some alpine areas span large latitudinal gradients, making the latitude centroid less representative of the entire region, we additionally included latitudinal range, defined as the difference in latitude between its northernmost and southernmost boundaries of each alpine area, as a linear fixed effect (Supplementary Data 1). The final formula looked like: log (1 + *richness*) ~ s (*latitude centroid*) + log (*latitudinal range*). We also tested modelling latitudinal range as a smooth term using thin plate regression splines (i.e., the default in mgcv::gam), but since this did not significantly improve the model, we opted for the simpler linear fixed effect instead. The GAM was fitted using normal (Gaussian) error distribution and Restricted Maximum Likelihood (REML), which optimises the selection of smoothing parameters and helps prevent overfitting. Model performance was evaluated based on explained deviance and significance of the predictors.

### Reporting summary

Further information on research design is available in the Nature Portfolio Reporting Summary linked to this article.

## Supplementary information

Supplementary Information

Descriptions of Additional Supplementary Files

Supplementary Data 1-4

Reporting Summary

Transparent Peer Review file

## Data Availability

All data generated and analysed in the study, including previously available datasets, are available on Zenodo (https://zenodo.org/records/20183570). The checklists for alpine mammals, birds, reptiles and vascular plants are available for download on Zenodo (https://zenodo.org/records/20183570) and through the GMBA Mountain Portal (https://mountainbiodiversity.org/). Note that all data made here available based on the version 1. of this dataset. In future, newer and refined versions may be available.

## Code availability

All code to produce the main results presented in this article is available as a Zenodo release on GitHub (https://zenodo.org/records/14925032).
