# Supplementary material for: Contrasting patterns of alpine biodiversity across mountains and taxa worldwide
Source: Nat Commun. 2026 Jul 3;17:9847. doi: 10.1038/s41467-026-75210-6 (PMC13578352; doi:10.1038/s41467-026-75210-6)
Supplement: Supplementary file 1 [file 41467_2026_75210_MOESM1_ESM.pdf]

Supplementary Information: Figures

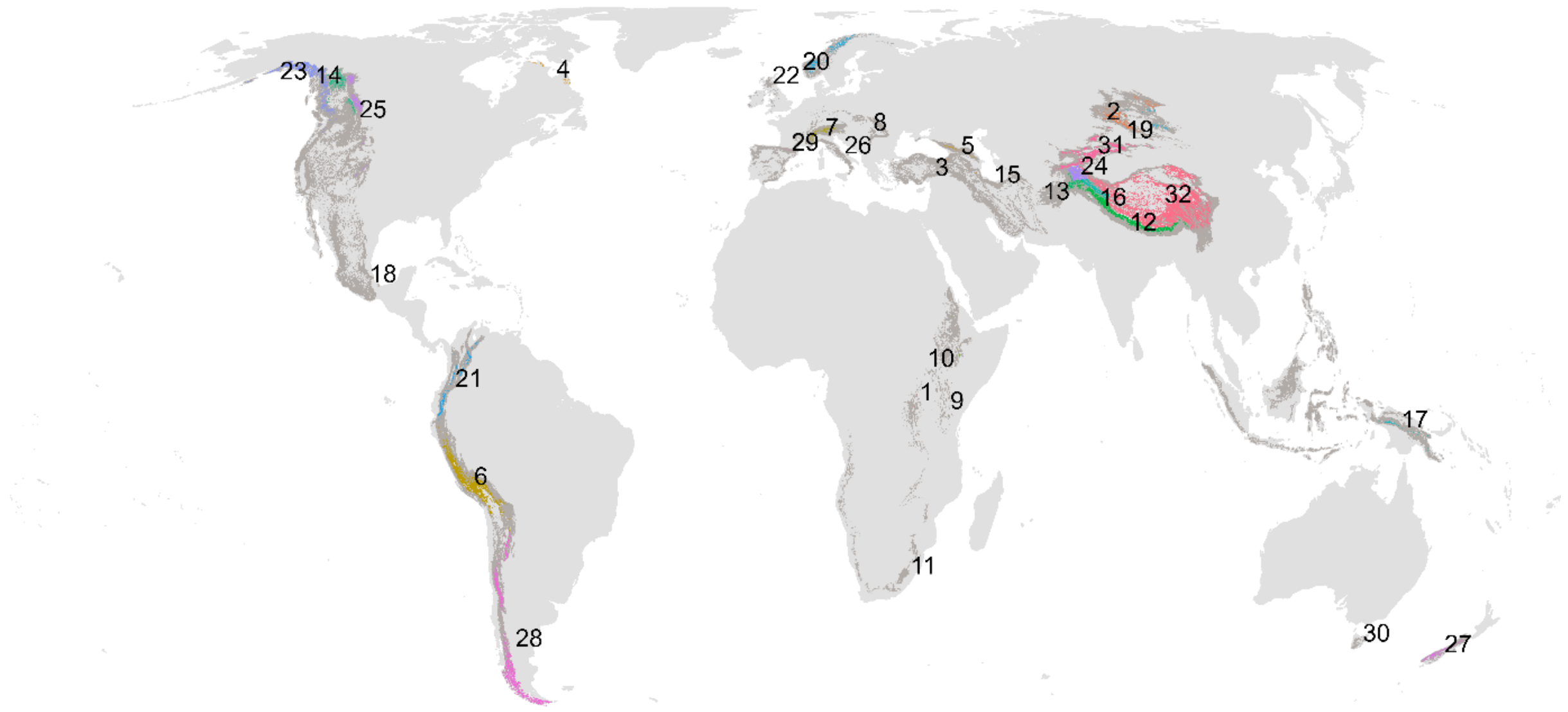

Supplementary Figure 1 | Mountain ranges containing climate-delineated alpine biome included in our dataset and analysis. Mountain ranges are shown in brown and follow mountain range delimitation by Snethlage et al. 2022 (GMBA version 02, Level 03). Numbers correspond to the mountain ranges used in this study and are detailed in Supplementary Information Table 1. Coloured areas represent the extent of alpine ecosystems within each mountain range.

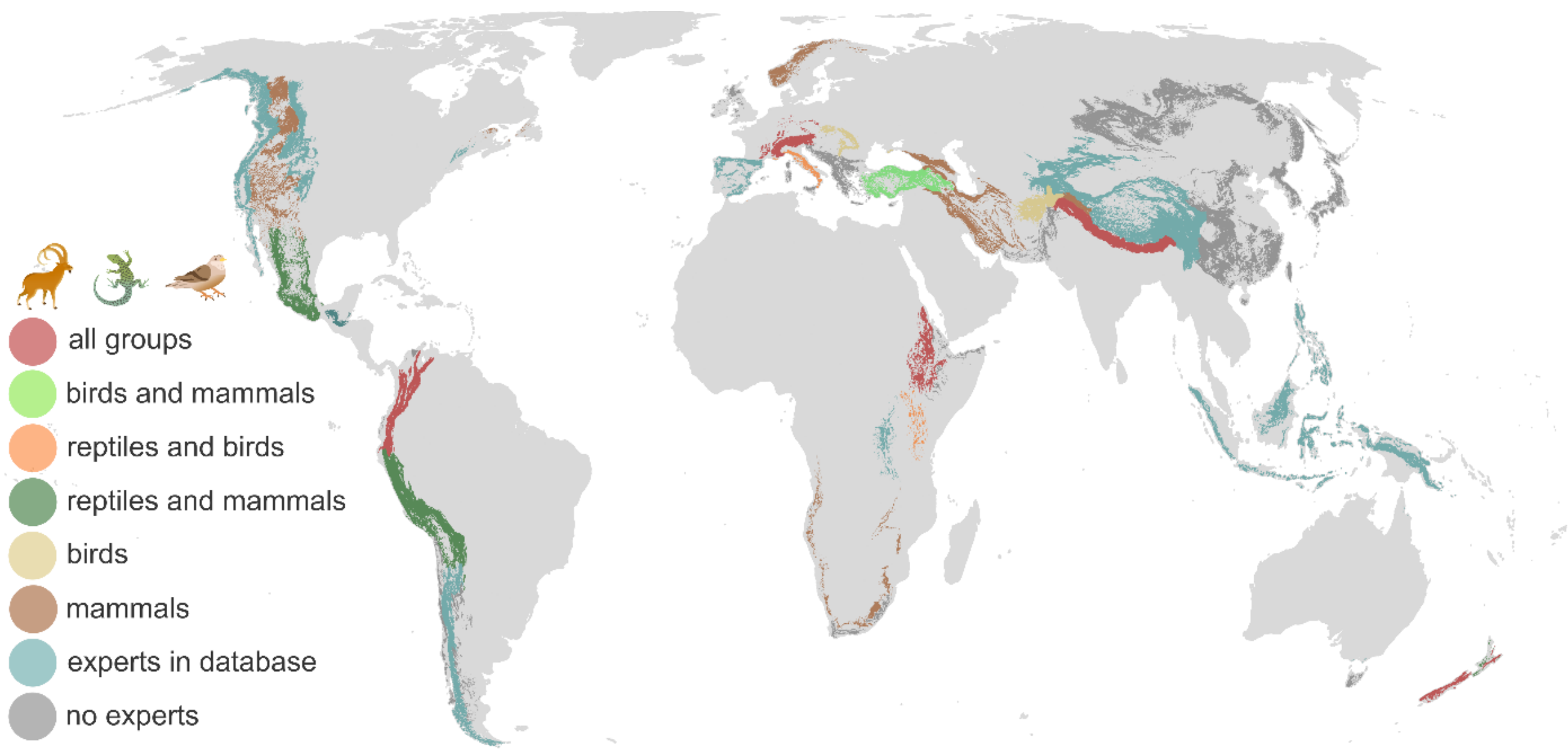

Supplementary Figure 2 | Global distribution of mountain ranges indicating presence and absence of validated vertebrate checklists. Colours indicate which vertebrate checklists have been validated by experts. Blue indicates mountain ranges where expert contacts are available, but checklists are yet to be validated. Grey denotes mountain ranges where we had no available expert contacts.

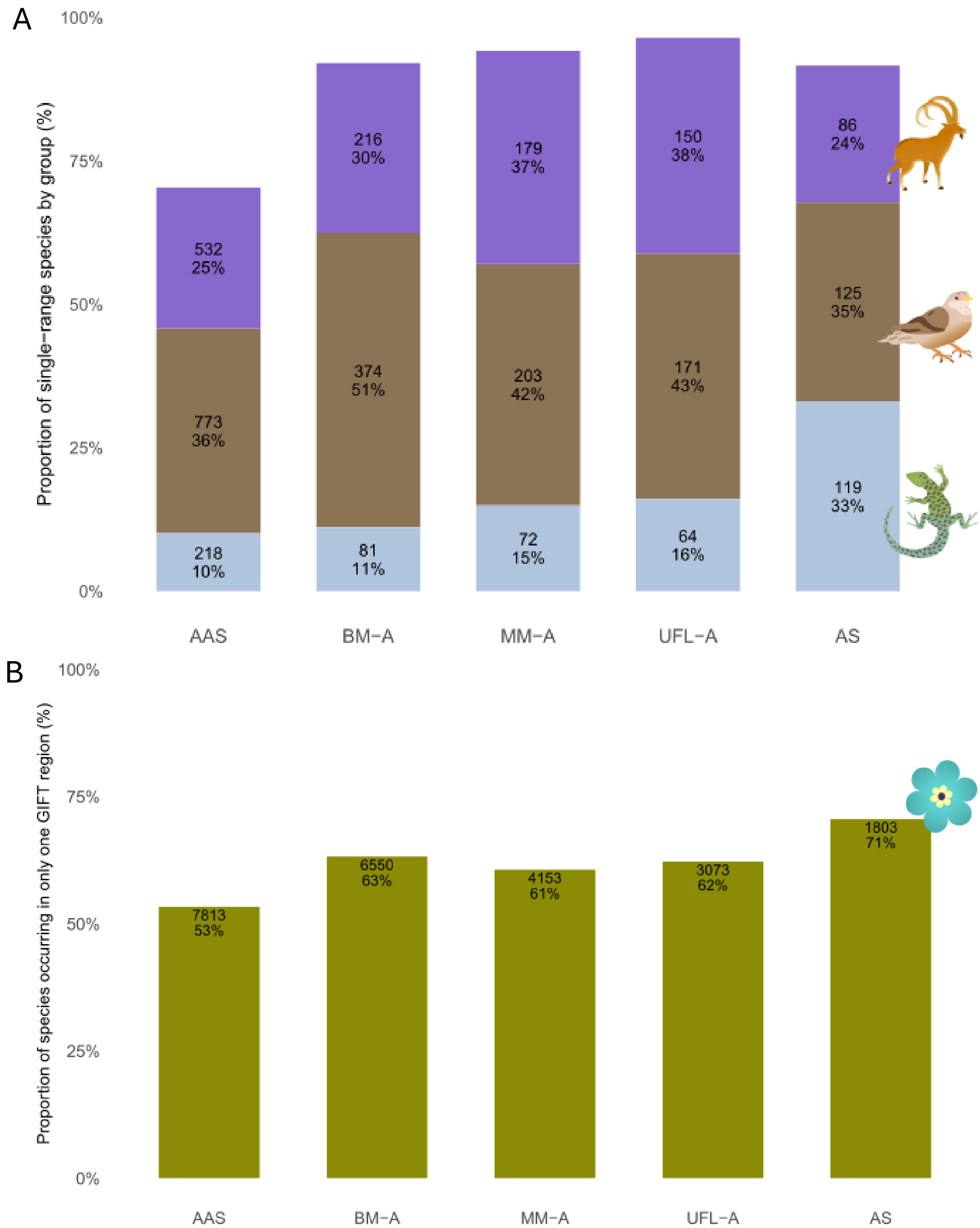

Supplementary Figure 3 | Proportion of (A) vertebrate species and (B) plant species within each alpine category that are restricted to a single mountain range (vertebrates) or a single GIFT region (plants). Numbers within bars indicate species counts; percentages indicate the proportion of single-range species. In panel A, coloured segments represent mammals (purple), birds (brown), and reptiles (blue). AAS = all alpine species, BM–A = broad montane–alpine species, MM–A = mid montane–alpine species, UFL–A = UFL–alpine species, and AS = alpine specialists.

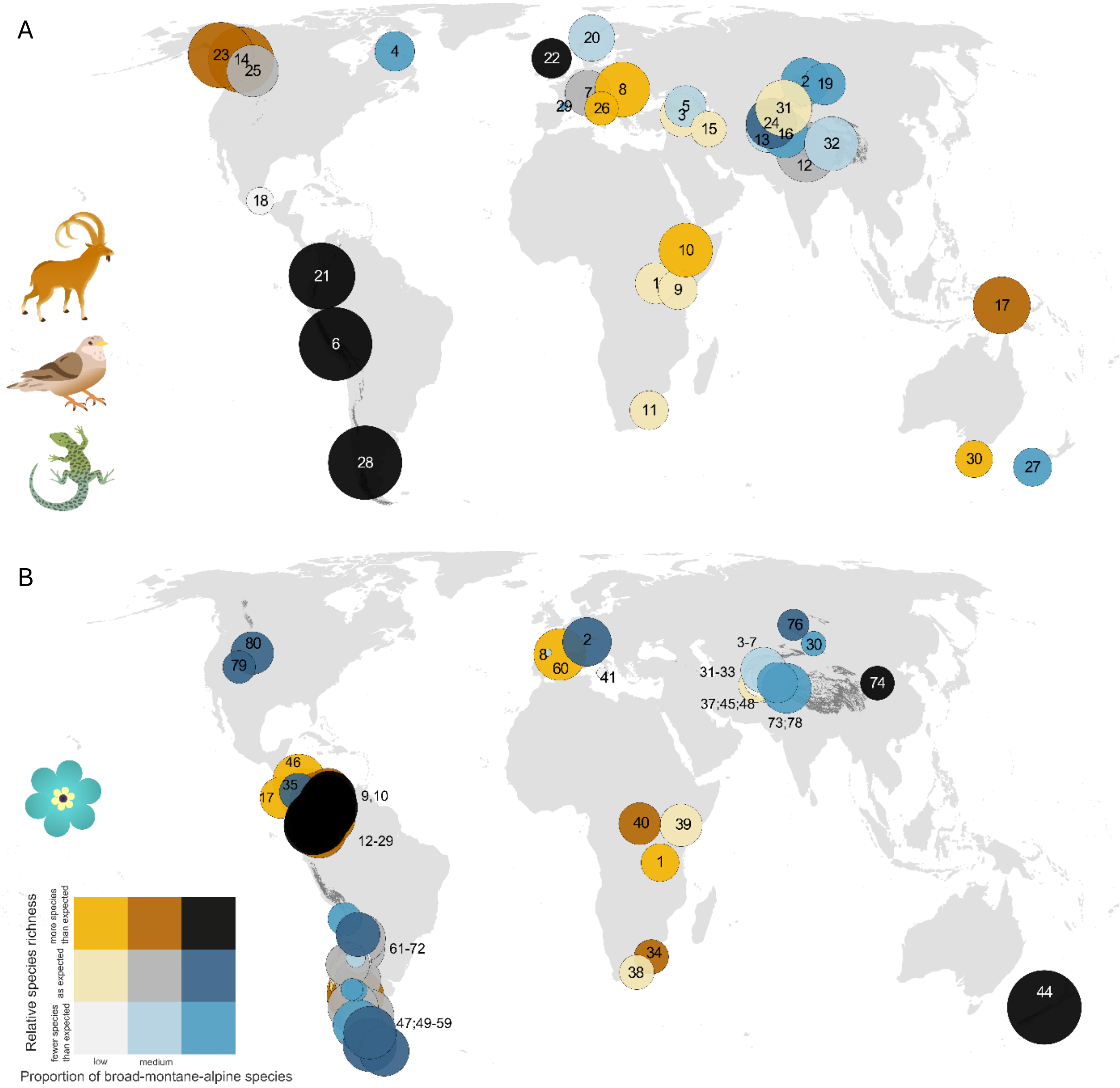

Supplementary Figure 4 | Absolute and relative richness for alpine vertebrates (A) and plants (B) and proportion of broad-montane-alpine species (i.e. elevational ranges fall within 6°C below the upper forest line). Bubble size represents the absolute richness of alpine species across all categories. Bubble colour indicates two factors related to the inset graph: (i) the proportion of broad-montane-alpine species along the X-axis and (ii) the richness of these species relative to area size along the Y-axis. Numbers correspond to mountain ranges for vertebrates and GIFT regions for plants, as detailed in Supplementary Data 1 and 2, respectively. See Fig. 2 for the full caption.

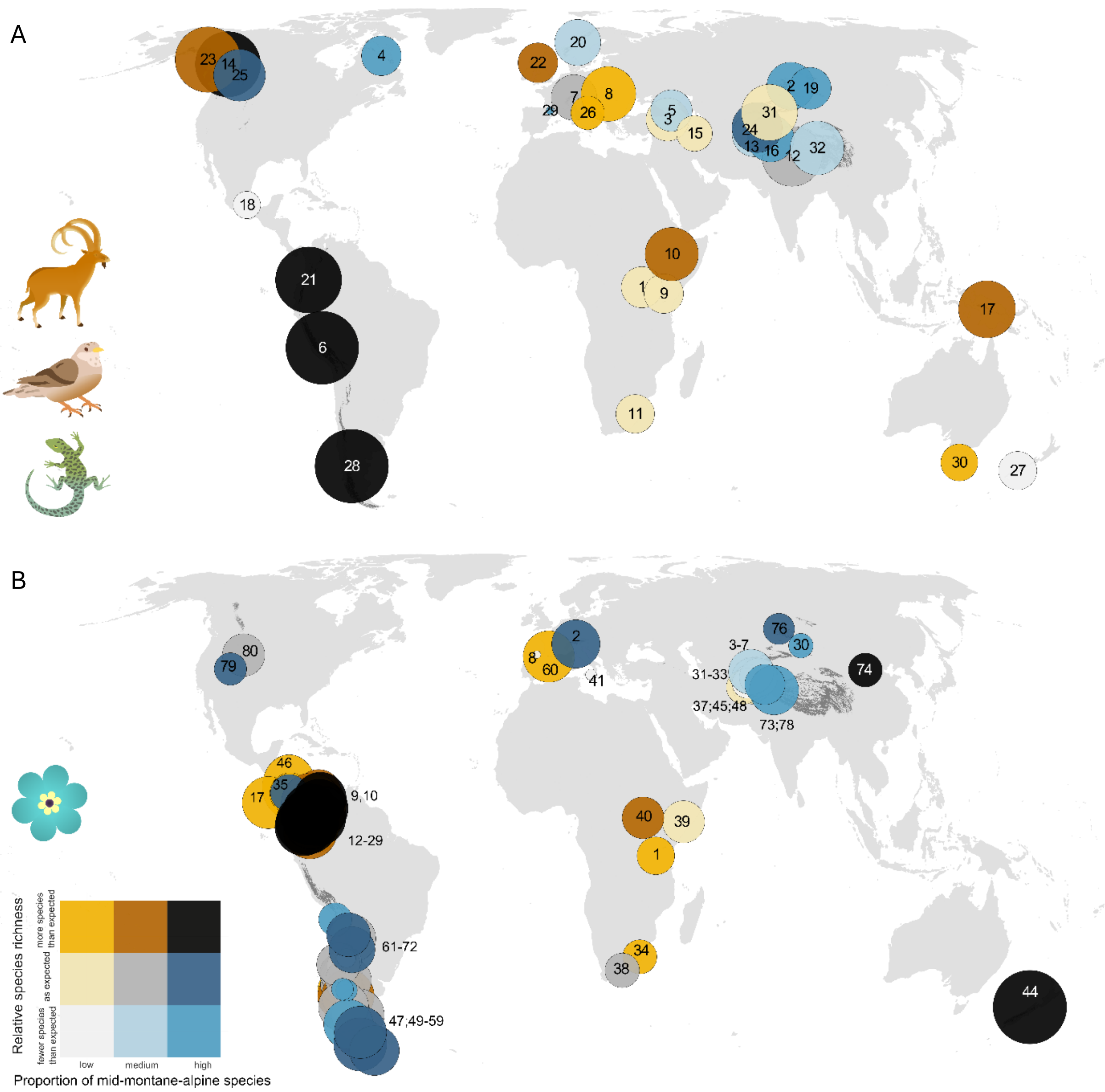

Supplementary Figure 5 | Absolute and relative richness for alpine vertebrates (A) and plants (B) and proportion of mid-montane-alpine species (i.e. elevational ranges fall within 4°C below the UFL). Bubble size represents the absolute richness of alpine species across all categories. Bubble colour indicates two factors related to the inset graph: (i) the proportion of mid-montane-alpine species along the X-axis and (ii) the richness of these species relative to area size along the Y-axis. Numbers correspond to mountain ranges for vertebrates and GIFT regions for plants, as detailed in Supplementary Data 1 and 2, respectively. See Fig. 2 for the full caption.

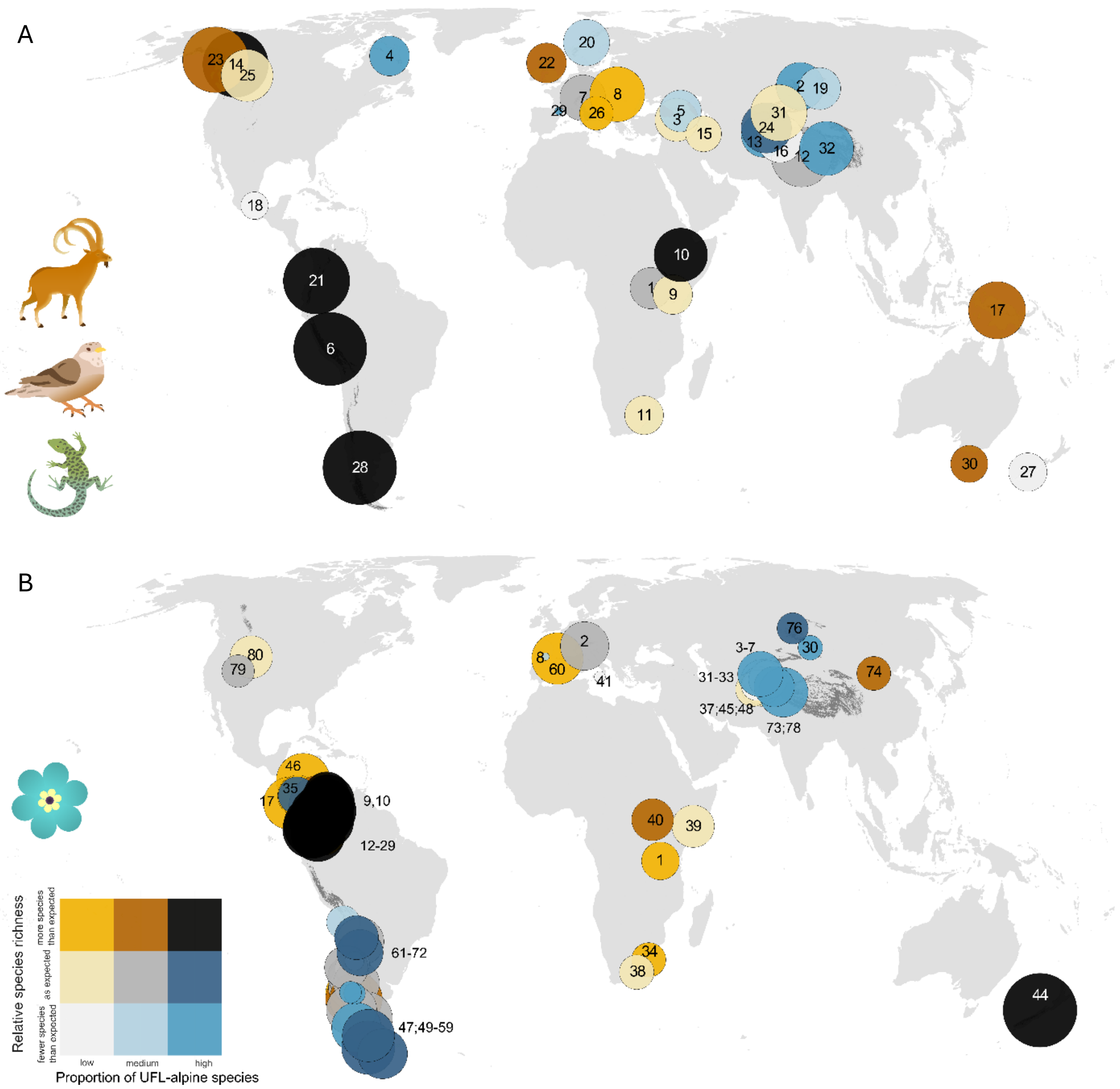

Supplementary Figure 6 | Absolute and relative richness for UFL-alpine (i.e. elevational ranges fall within 2°C below the UFL) vertebrates (A) and plants (B). Bubble size represents the absolute richness of alpine species across all categories. Bubble colour indicates two factors related to the inset graph: (i) the proportion of UFL-alpine species along the X-axis and (ii) the richness of these species relative to area size along the Y-axis. Numbers correspond to mountain ranges for vertebrates and GIFT regions for plants, as detailed in Supplementary Data 1 and 2, respectively. See Fig. 2 for the full caption.

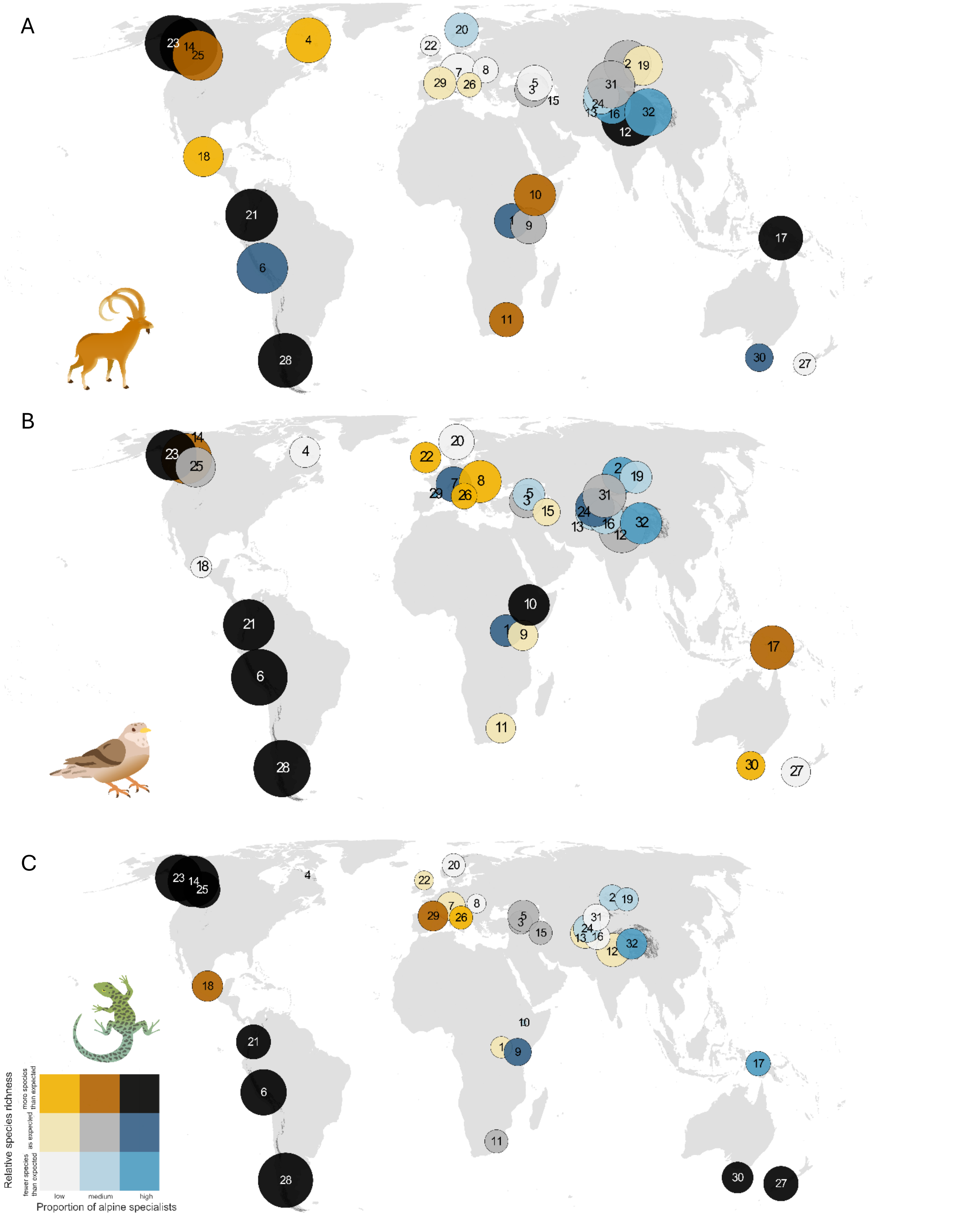

Supplementary Figure 7 | Global distribution of absolute and relative richness and proportion of alpine specialists for individual vertebrate groups of mammals (A), birds (B), and reptiles (C). Bubble size represents the absolute richness of alpine species across all categories. Bubble colour indicates two factors related to the inset graph: (i) the proportion of alpine specialists along the X-axis and (ii) the richness of these species relative to area size along the Y-axis. Numbers

correspond to mountain ranges for vertebrates and GIFT regions for plants, as detailed in Supplementary Data Tables 1 and 2, respectively. See Fig. 2 for the full caption.

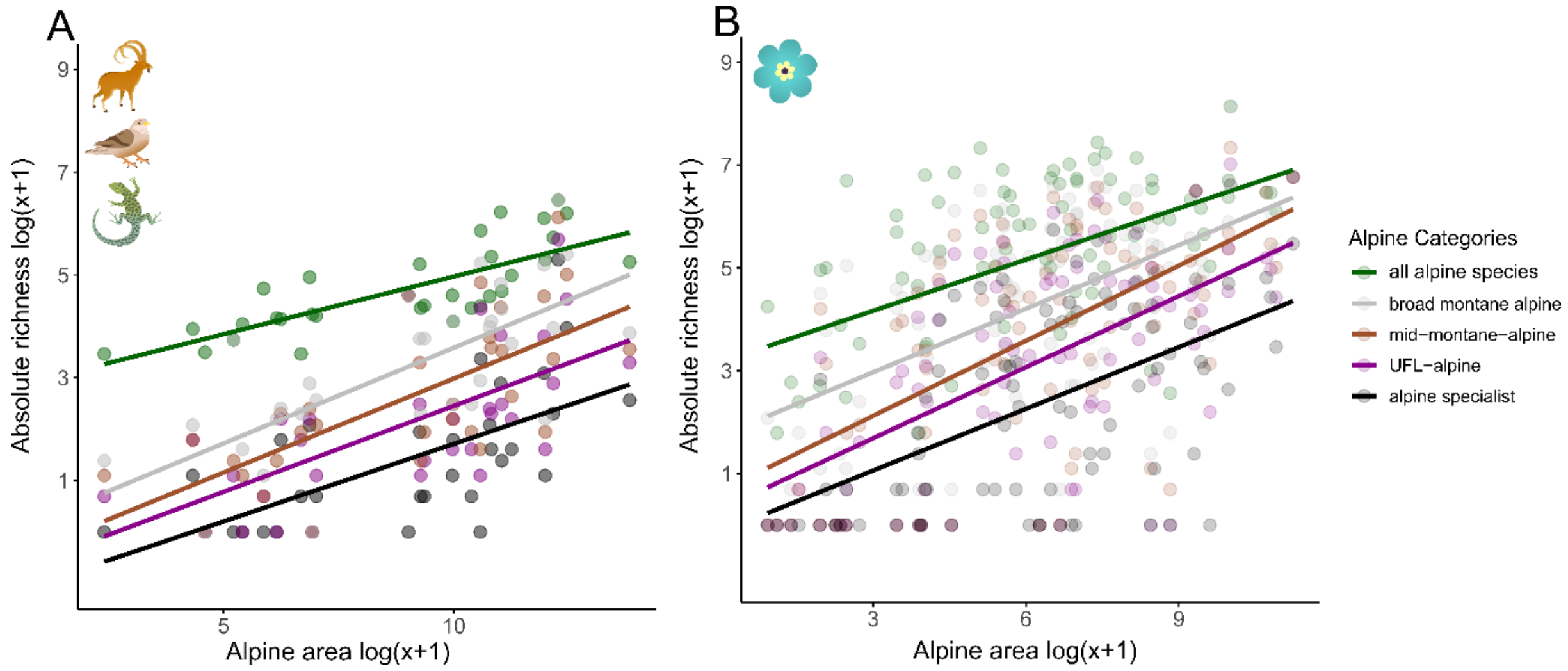

Supplementary Figure 8 | Species-Area Relationships (SAR) for plants (A) and vertebrates (B) across alpine categories. Lines represent linear regressions between alpine area and absolute richness. Points represent individual alpine regions (plants: n=80 regions; vertebrates: n=32 regions). Colours indicate alpine categories: all alpine species (green), broad montane–alpine (brown), mid-montane–alpine (purple), UFL–alpine (purple), and alpine specialists (black). The Z-values (slopes of the SAR) derived for each category are: all alpine species (vertebrates: 0.23, plants: 0.31), alpine specialists (vertebrates: 0.30, plants: 0.38), UFL-alpine (vertebrates: 0.34, plants: 0.42), mid-montane-alpine (vertebrates: 0.38, plants: 0.46), and broad montane alpine (vertebrates: 0.37, plants: 0.40). All SAR slopes were significant (vertebrates: (t=4.28–5.43, df=28–32, all p<0.001, R^2=0.38–0.50); plants:5.13-6.74, df= 84, all p<0.001, R^2=0.23-0.35).
