# Supplementary material for: Contrasting patterns of alpine biodiversity across mountains and taxa worldwide
Source: Nat Commun. 2026 Jul 3;17:9847. doi: 10.1038/s41467-026-75210-6 (PMC13578352; doi:10.1038/s41467-026-75210-6)
Supplement: Supplementary file 2 [file 41467_2026_75210_MOESM2_ESM.pdf]

Description of Additional Supplementary Files

Supplementary Data 1: Details of alpine areas within each mountain range: mean elevation of the upper forest line (UFL), alpine area, location (centroid longitude, latitude and latitudinal range).

Supplementary Data 2: Details of alpine areas within each GIFT region: alpine area (km2), mean elevation of the upper forest line (UFL), location (centroid longitude and latitude), and the corresponding reference to each GIFT region.

Supplementary Data 3: Absolute richness (aR) and relative richness (rR) for mammals, birds and reptiles across mountain ranges and alpine categories.

Supplementary Data 4: Absolute richness (aR) and relative richness (rR) for vascular plants across GIFT regions and alpine categories.
