# Supplementary material for: Contrasting patterns of alpine biodiversity across mountains and taxa worldwide
Source: Nat Commun. 2026 Jul 3;17:9847. doi: 10.1038/s41467-026-75210-6 (PMC13578352; doi:10.1038/s41467-026-75210-6)
Supplement: Supplementary file 3 [file 41467_2026_75210_MOESM4_ESM.pdf]

nature portfolio

Corresponding author(s): Lotta Schultz

Last updated by author(s): May 22, 2026

Reporting Summary

Nature Portfolio wishes to improve the reproducibility of the work that we publish. This form provides structure for consistency and transparency in reporting. For further information on Nature Portfolio policies, see our Editorial Policies and the Editorial Policy Checklist.

Statistics

For all statistical analyses, confirm that the following items are present in the figure legend, table legend, main text, or Methods section.

n/a	Confirmed	
☐	☒	The exact sample size (*n*) for each experimental group/condition, given as a discrete number and unit of measurement
☒	☐	A statement on whether measurements were taken from distinct samples or whether the same sample was measured repeatedly
☐	☒	The statistical test(s) used AND whether they are one- or two-sided<br>*Only common tests should be described solely by name; describe more complex techniques in the Methods section.*
☒	☐	A description of all covariates tested
☐	☒	A description of any assumptions or corrections, such as tests of normality and adjustment for multiple comparisons
☐	☒	A full description of the statistical parameters including central tendency (e.g. means) or other basic estimates (e.g. regression coefficient) AND variation (e.g. standard deviation) or associated estimates of uncertainty (e.g. confidence intervals)
☐	☒	For null hypothesis testing, the test statistic (e.g. *F*, *t*, *r*) with confidence intervals, effect sizes, degrees of freedom and *P* value noted<br>*Give P values as exact values whenever suitable.*
☒	☐	For Bayesian analysis, information on the choice of priors and Markov chain Monte Carlo settings
☒	☐	For hierarchical and complex designs, identification of the appropriate level for tests and full reporting of outcomes
☒	☐	Estimates of effect sizes (e.g. Cohen's *d*, Pearson's *r*), indicating how they were calculated

Our web collection on statistics for biologists contains articles on many of the points above.

Software and code

Policy information about availability of computer code

Data collection

Data were compiled from multiple published databases and literature sources (see Data section). All data processing and compilation were done in R version 4.4.2. Plant data were collected from GIFT (Global Inventory of Flora and Traits, version 3.2) and accessed through the GIFT R package (version 1.2.0).
Data wrangling and cleaning were performed primarily using the tidyverse suite of packages (version 2.0.0).

GIFT—An R package to access the Global Inventory of Floras and TraitsDenelle, P., Weigelt, P. & Kreft, H. GIFT—An R package to access the Global Inventory of Floras and Traits. Methods in Ecology and Evolution 14, 2738–2748 (2023).

Wickham H, Averick M, Bryan J, Chang W, McGowan LD, François R, Grolemun G, Haye A, Henr L, Heste J, Kuh M, Pederse TL, Mille E, Bach SM, Müll K, Oo ,J, Robins ,D, Seid ,DP, Spi ,V, Takahas ,K, Vaugh ,D, Wil ,C, W ,K, Yutani ,H (2019). Welcome to the tidyverse. Journal of Open Source Software, https://doi.org/10.21105/joss.01686.

R Core Team. R: A language and environment for statistical computing. R Foundation for Statistical Computing, Vienna, Austria. (2023).

Data analysis	All code written in R version 4.4.2 . All code to produce the main results presented in this article is available as a Zenodo release on GitHub (https://doi.org/10.5281/zenodo.14925032).<br>We used the mgcv R package (version 1.8.42) to fit Generalized Additive Models (GAMs) to identfiy latitudinal trends in absolute and relative richness for vertebrates and plants<br>Wood, S. mgcv: Mixed GAM Computation Vehicle with Automatic Smoothness Estimation. (2023).<br>R Core Team. R: A language and environment for statistical computing. R Foundation for Statistical Computing, Vienna, Austria. (2023).

For manuscripts utilizing custom algorithms or software that are central to the research but not yet described in published literature, software must be made available to editors and reviewers. We strongly encourage code deposition in a community repository (e.g. GitHub). See the Nature Portfolio guidelines for submitting code & software for further information.

Data

Policy information about availability of data

All manuscripts must include a data availability statement. This statement should provide the following information, where applicable:

- Accession codes, unique identifiers, or web links for publicly available datasets
- A description of any restrictions on data availability
- For clinical datasets or third party data, please ensure that the statement adheres to our policy

All data generated and analyzed in the study, including previously available datasets are available on Zenodo (LINK). The checklists for alpine mammals, birds, reptiles and vascular plants are available for download on Zenodo (https://doi.org/10.5281/zenodo.20183570) and through the GMBA Mountain Portal (LINK). Note that all data made here available based on the version 1. of this dataset. In future, newer and refined versions may be available.

The raw data was obtained from the following sources:

Mammal Data
Distribution data: Mammal Diversity Database (MDD; version 1.11; accessed May, 2023)

Data citation: Mammal Diversity Database. (2023). Mammal Diversity Database (1.11) [Data set]. Zenodo. https://doi.org/10.5281/zenodo.7830771

Paper citation: Marsh, C. J., Sica, Y. V., Burgin, C. J., Dorman, W. A., Anderson, R. C., et al. (2022). Expert range maps of global mammal distributions harmonised to three taxonomic authorities. Journal of Biogeography, 49, 979–992. https://doi.org/10.1111/jbi.14330

Elevation data: Elevational data were compiled from the Handbook of the Mammals of the World series (Wilson & Mittermeier, 2009–2019) using the machine-readable extraction provided by J. H. Poelen (accessed June, 2023): jhpoelen/hmw GitHub repository

Reptile Data
Distribution data: Global Assessment of Reptile Distributions (GARD; version 1.7, accessed June, 2023)

Data citation: Roll, Uri; Meiri, Shai (2022). GARD 1.7 - updated global distributions for all terrestrial reptiles [Dataset]. Dryad. https://doi.org/10.5061/dryad.9cnp5hqmb

Paper citation: Roll, U., Feldman, A., Novosolov, M., Allison, A., Bauer, A. M., Bernard, R., Böhm, M., Castro-Herrera, F., Chirio, L., Collen, B., Colli, G. R., et al. (2017). The global distribution of tetrapods reveals a need for targeted reptile conservation. Nature Ecology & Evolution, 1, 1677–1682. https://doi.org/10.1038/s41559-017-0332-2 and de Oliveira Caetano, G. H., et al. (2022). Automated assessment reveals that the extinction risk of reptiles is widely underestimated across space and phylogeny. PLOS Biology, 20(5), e3001544. https://doi.org/10.1371/journal.pbio.3001544

Elevation data: SquamBase (version 1.0, accessed June, 2023 (provided by S. Meiri prior to publication and accessed June 2023); Meiri, 2024) Data citation: Meiri, Shai (2024). SquamBase 1.0 – a dataset of squamate species traits [Dataset]. Dryad. https://doi.org/10.5061/dryad.76hdr7t3b

Paper citation: Meiri, S. (2024). SquamBase—A database of squamate (Reptilia: Squamata) traits. Global Ecology and Biogeography, 33(4), e13812. https://doi.org/10.1111/geb.13812

Bird Data
Distribution data: BirdLife International Data Zone (version 2022.2, accessed May 2023)

BirdLife International and Handbook of the Birds of the World (2022) Bird species distribution maps of the world. Version 2022.2. Available at http://datazone.birdlife.org/species/requestdis.

Elevation data: Quintero, I., Jetz, W. Global elevational diversity and diversification of birds. Nature 555, 246–250 (2018). https://doi.org/10.1038/nature25794 (Supplementary Table 1 version of record, 2018, accessed June 2023)

Plant Data
Regional plant checklists: Global Inventory of Floras and Traits (GIFT) database (version 3.0, access provided by P. Denelle prior to publication and accessed April 2023)

Weigelt, P., König, C. & Kreft, H. (2020) GIFT - A Global Inventory of Floras and Traits for macroecology and biogeography. Journal of Biogeography, 47, 16-43. DOI: 10.1111/jbi.13623

Mountain Data
Mountain inventory: GMBA Mountain Inventory (version 2, accessed April 2023)

Data citation: Snethlage, M.A., Geschke, J., Spehn, E.M., Ranipeta, A., Yoccoz, N.G., Körner, Ch., Jetz, W., Fischer, M. & Urbach, D. (2022) GMBA Mountain Inventory v2. GMBA-EarthEnv. https://doi.org/10.48601/earthenv-t9k2-1407

Paper citation: Snethlage, M.A., Geschke, J., Spehn, E.M., Ranipeta, A., Yoccoz, N.G., Körner, Ch., Jetz, W., Fischer, M. & Urbach, D. (2022). A hierarchical inventory of the world's mountains for global comparative mountain science. Nature Scientific Data. https://doi.org/10.1038/s41597-022-01256-y

Research involving human participants, their data, or biological material

Policy information about studies with human participants or human data. See also policy information about sex, gender (identity/presentation), and sexual orientation and race, ethnicity and racism.

Reporting on sex and gender	*Use the terms sex (biological attribute) and gender (shaped by social and cultural circumstances) carefully in order to avoid confusing both terms. Indicate if findings apply to only one sex or gender; describe whether sex and gender were considered in study design; whether sex and/or gender was determined based on self-reporting or assigned and methods used. Provide in the source data disaggregated sex and gender data, where this information has been collected, and if consent has been obtained for sharing of individual-level data; provide overall numbers in this Reporting Summary. Please state if this information has not been collected. Report sex- and gender-based analyses where performed, justify reasons for lack of sex- and gender-based analysis.*
Reporting on race, ethnicity, or other socially relevant groupings	*Please specify the socially constructed or socially relevant categorization variable(s) used in your manuscript and explain why they were used. Please note that such variables should not be used as proxies for other socially constructed/relevant variables (for example, race or ethnicity should not be used as a proxy for socioeconomic status). Provide clear definitions of the relevant terms used, how they were provided (by the participants/respondents, the researchers, or third parties), and the method(s) used to classify people into the different categories (e.g. self-report, census or administrative data, social media data, etc.) Please provide details about how you controlled for confounding variables in your analyses.*
Population characteristics	*Describe the covariate-relevant population characteristics of the human research participants (e.g. age, genotypic information, past and current diagnosis and treatment categories). If you filled out the behavioural & social sciences study design questions and have nothing to add here, write "See above."*
Recruitment	*Describe how participants were recruited. Outline any potential self-selection bias or other biases that may be present and how these are likely to impact results.*
Ethics oversight	*Identify the organization(s) that approved the study protocol.*

Note that full information on the approval of the study protocol must also be provided in the manuscript.

Field-specific reporting

Please select the one below that is the best fit for your research. If you are not sure, read the appropriate sections before making your selection.

☐ Life sciences ☐ Behavioural & social sciences ☒ Ecological, evolutionary & environmental sciences

For a reference copy of the document with all sections, see nature.com/documents/nr-reporting-summary-flat.pdf

Ecological, evolutionary & environmental sciences study design

All studies must disclose on these points even when the disclosure is negative.

Study description	This study analyzes global patterns of alpine biodiversity. The study provides a dataset for alpine birds, mammals, reptiles and vascular plants and compares biodiversity patterns across taxonomic groups, mountain ranges worldwide and different categories of species elevational specialization.
Research sample	The study analyzes species richness of 32 mountain ranges around the world that contain alpine biome. 711 mammals, 1,173 birds, 281 reptiles and 14,649 vascular plants occuring within the alpine zone of these mountain ranges are included in the analyses.
Sampling strategy	complete analyses of mountain ranges with alpine biome.
Data collection	All data used for the analyses is previously published and freely available (see Data section).
Timing and spatial scale	Spatial scale is global. Raw species distribution data is used from the most up to date databases (all published between 2021-2024) and refined by current expert knowledge.
Data exclusions	No data were excluded
Reproducibility	All data generated and analyzed in the study, including previously available datasets are available on Zenodo https://doi.org/10.5281/zenodo.20183570 The checklists for alpine mammals, birds, reptiles and vascular plants are available for download on Zenodo https://doi.org/10.5281/zenodo.20183570 and through the GMBA Mountain Portal https://mountainbiodiversity.org/ Note that all data made here available based on the version 1. of this dataset. In future, newer and refined versions may be available. All code to produce the main results presented in this article is available as a Zenodo release on GitHub (https://doi.org/10.5281/zenodo.14925032)

Randomization	NA
Blinding	NA

Did the study involve field work? ☐ Yes ☒ No

Reporting for specific materials, systems and methods

We require information from authors about some types of materials, experimental systems and methods used in many studies. Here, indicate whether each material, system or method listed is relevant to your study. If you are not sure if a list item applies to your research, read the appropriate section before selecting a response.

Materials & experimental systems

n/a	Involved in the study
☒	☐ Antibodies
☒	☐ Eukaryotic cell lines
☒	☐ Palaeontology and archaeology
☒	☐ Animals and other organisms
☒	☐ Clinical data
☒	☐ Dual use research of concern
☒	☐ Plants

Methods

n/a	Involved in the study
☒	☐ ChIP-seq
☒	☐ Flow cytometry
☒	☐ MRI-based neuroimaging

Plants

Seed stocks	*Report on the source of all seed stocks or other plant material used. If applicable, state the seed stock centre and catalogue number. If plant specimens were collected from the field, describe the collection location, date and sampling procedures.*
Novel plant genotypes	*Describe the methods by which all novel plant genotypes were produced. This includes those generated by transgenic approaches, gene editing, chemical/radiation-based mutagenesis and hybridization. For transgenic lines, describe the transformation method, the number of independent lines analyzed and the generation upon which experiments were performed. For gene-edited lines, describe the editor used, the endogenous sequence targeted for editing, the targeting guide RNA sequence (if applicable) and how the editor was applied.*
Authentication	*Describe any authentication procedures for each seed stock used or novel genotype generated. Describe any experiments used to assess the effect of a mutation and, where applicable, how potential secondary effects (e.g. second site T-DNA insertions, mosiacism, off-target gene editing) were examined.*
