# Supplementary material for: Contrasting patterns of alpine biodiversity across mountains and taxa worldwide
Source: Nat Commun. 2026 Jul 3;17:9847. doi: 10.1038/s41467-026-75210-6 (PMC13578352; doi:10.1038/s41467-026-75210-6)
Supplement: Supplementary file 4 [file 41467_2026_75210_MOESM5_ESM.pdf]

nature portfolio

Peer Review File

Contrasting patterns of alpine biodiversity across mountains and taxa worldwide

Corresponding Author: Ms Lotta Schultz

This file contains all reviewer reports in order by version, followed by all author rebuttals in order by version.

Version 0:

Reviewer comments:

Reviewer #1

(Remarks to the Author)
This is a very impressive study, and very valuable global product from the GMBA Mountain Portal and other databases. Detailed empirical studies of significant but fragmented systems - such as the alpine biome - across the globe are scarce. This paper has a strength of being truly globally representative, and creates a baseline for additional studies and understanding on alpine areas globally. It speaks to global conventions and policy, with mountain-specific application for national/transboundary interest and action.

Abstract
I would add to the last sentence "suggesting diverse historical biogeographies of alpine areas". Something the result show is that alpine areas differ tremendously between them in terms of richness, as well richness between phyla. Area explains some it, but as aluded to, historical biogeography plays a tremendous role in explaining this.

Main Text
1. p.3, l.59 - change "thrive" to "occur".
2. p.8-9, ll.236-245 - important comments on historical biogeographies. The paper does lend itself to more interpretation perhaps on this. (Miocene)Pliocene-Pleistocene species pumps from climatic oscillations, uplift events, etc., and the data lends itself to potentially powerful global theory of island biogeography application. The background extinction rate in alpine areas compared to global change extinction rate/projected would add a powerful impetus to the paper's impact.
3. p.10, ll.280-282 - speaks to 2. above as well. e.g. Lower plant richness in the Drakensberg for instance is likely due to the alpine area only being "lower alpine", with an elevation depth of only c.400 m, due to the ancient, planed Gondwana land-surface on the summit (low summit relief, and limited topographic heterogeneity), probably resulting in a higher background extinction rate in an area with high climatic variability and alpine specialists needing to re-evolve post warm events.
4. p.10, "data limitations" etc. - personally I feel this section could be more punchy. The paper lays a good foundation, but next steps should be more clear - opportunities should be clearly outlined, and the paper could position itself as a "communication" more strongly for better support and action (data collection, policy impact, etc.). Make the strengths and weakness of the paper more succinct (less rambling), and outline what "could be" building on this data.

Methods:
1. Were species extinct in historical/near-historical times included? This can affect some results, e.g. perhaps in New Zealand (Moas, other land birds)? Extirpated species from some alpine areas but still present in others e.g. Lammergeier? This may change some data results and also emphasize human impact on alpine species (sensu Global Change not just climate change).
2. I would suggest corroborating the delimitation of alpine areas used in the methods with a climatic one, e.g. 6oC isotherm. This would give the delimitations more weight and justification, and or suggest modifications.
3. The latitudinal question is NB, and well-integrated into the study. However perhaps it should be mentioned that although climate is "similar" across alpine areas (e.g. mean ann temp 6oC), latitude plays a large role in local abiotic includ. climate processes due to daylight length, and daily temp fluctuations vs seasonal temp fluctuations. Tropical alpines have "summer every day and winter every night" with little insolation variability, while temperate and polar alpines have extended seasonal insolation differences driving highly variable daylight lengths and seasonal temperature differences.
4. Re. birds, long-distance migrants are mentioned. Local and regional migrants are worth a mention, e.g. alpine breeding endemics that disperse seasonally to other areas within-continent, or to lower elevations nearby.

Figures etc.
1. Extended Data Table 3 - standardise use of capitals and spacing in column headings.
2. Other figures and diagrams suitable etc.

(Remarks on code availability)

Reviewer #2

(Remarks to the Author)
I co-reviewed this manuscript with one of the reviewers who provided the listed reports. This is part of the Nature Communications initiative to facilitate training in peer review and to provide appropriate recognition for Early Career Researchers who co-review manuscripts.

(Remarks on code availability)
The code was tested and it runs very well. The instructions are adequate and easy to follow. All required packages installed without any challenges.

Reviewer #3

(Remarks to the Author)
STRENGTHS:
This study seeks to enhance our comparative understanding of alpine diversity by providing a "comprehensive, expert-validated dataset" on global alpine biodiversity for several, broad taxonomic groups (vascular plants, mammals, birds, and reptiles). There is absolutely a need for more data in this field and the authors are providing critical information by helping to fulfill that need. One of the main strengths of a dataset such as this is the "expert-validation" aspect, which is often missing from these types of big-data analyses. Another strength is that the authors parse species into alpine generalist and specialist categories, which has been noted in the literature as a method to obtain more biologically-relevant categories.

The authors' results corroborate and build off previous literature examining broad spatial biodiversity patterns among alpine species. They find that many alpine species are "super-specialists," often confined to a single mountain range, which underscores the authors' assertions regarding the pivotal ecological role these ecosystems play and their importance for consideration in conservation planning.

Consistent with previous research, the authors find that Neotropical alpine biomes are biodiversity hotspots with a high degree of specialists and higher latitude alpine regions are less biodiverse with proportionally more generalist species. However, they also astutely illustrate and explain the decoupling of alpine biodiversity patterns from any traditional latitudinal gradient, offering a nice comparison of latitudinal patterns when climatic factors are more uniform. This enhances our overall understanding of alpine biodiversity as well as the underlying mechanisms that drive the traditional latitudinal diversity gradient. The authors are sensitive to the historical factors that likely have driven these overall patterns (phylogenetic as well as climatic and geologic).

WEAKNESSES/ CONSIDERATIONS:
I think there are critical science philosophy questions to consider about whether we, as researchers, really should expect one study to "do it all" in this manner and be able to provide a comprehensive dataset for such disparate groups – or even whether such data are comparable across these groups – however that is not a criticism of the authors approach or goals, but a reflection of the overall culture in this field.

Nevertheless, I caution against calling this (or any) dataset "comprehensive." No matter how extensive or carefully curated, there will always be gaps. The authors do a good job of explaining the gaps in their own dataset, even as they call it comprehensive, for example citing the artefacts in their reptile data. Additionally, as research continues, current datasets always give way to new discoveries and refined methodologies, making old datasets obsolete very rapidly. The authors have curated a very refined and thoughtful comparative dataset, and I think it suffices for them to refer to it as such. Calling it "comprehensive" may invite criticisms from other scientists where there did not need to be any.

Overall, this study has contributed greatly and built a very nice dataset that is solid and usable by the community at large. However, although I truly enjoy this paper and its presentation, I am still left wondering "what's the main takeaway"? What is the big picture message I should have now that I didn't have before and what has the community learned from this that it did not already know?

The biggest finding seems to be the latitudinal patterns of biodiversity (or lack thereof). At the same time, though, the authors main framing in the abstract and introduction seems to highlight the conservation and biodiversity preservation aspect. I would like to see the authors tie their results to their big picture message more and give the readers a clear take-home and assessment of the import of the results overall for our understanding of alpine biodiversity. Right now, the main message seems to be, essentially, that there is a lot of heterogeneity, however we already know that is the norm for biological communities. So, what should we be doing with this heterogeneity? How do we apply that knowledge when thinking about conservation and safeguarding alpine ecosystems?

METHODS:
L363: Regarding a UFL vs treeline approach, was there any attempt made to see if the UFL approach outlined here produced very different results from a treeline approach? This could be done with just those areas for which the treeline approach can be implemented relatively easily as a validation check. If the two methods do not produce substantially different results, I think that is worth knowing/ stating for the future as the authors' UFL approach is computationally much easier.

L438: This is probably the single greatest strength of this dataset and I think the authors have done a really great job of pulling this off for such a wide variety of broad taxonomic groups.

L473: I agree with the authors here that this approach, though necessary, should be interpreted with caution. Particularly in cases where important inferences are being made solely based on this approach.

L493: It is unclear to me from this description and Fig. 1 if experts also validated the vascular plant portion of the dataset. If not, my question would be why not? Was it not needed due to the quality of the data or was it that experts were not sought out.

L557: For panels C/D, I find the gray shading hard to follow. That might be intentional since no strong pattern is observed, but I would prefer a different color scheme.

(Remarks on code availability)
I reviewed the GitHub broadly, not necessarily line by line in each file of code. However, overall I think this is an excellent repository and resource for the community. It is well organized and documented, appears to follow best practices, and there are tutorials and other training resources to allow users of all experience levels to use the provided code.

Version 1:

Reviewer comments:

Reviewer #1

(Remarks to the Author)
The authors have addressed my comments to my satisfaction. I recommend the paper for acceptance by the journal.

(Remarks on code availability)
NA

Reviewer #2

(Remarks to the Author)
I co-reviewed this manuscript with one of the reviewers who provided the listed reports. This is part of the Nature Communications initiative to facilitate training in peer review and to provide appropriate recognition for Early Career Researchers who co-review manuscripts.

(Remarks on code availability)
The code was not reviewed this time because the last time it was reviewed it was very well done. Since this was not reviewed it is not necessary to review it again.

Reviewer #3

(Remarks to the Author)
The authors appear to have integrated all the requested revisions. They have also thoughtfully addressed all the conceptual suggestions and critiques on framing and "big picture" messaging of the manuscript. I feel satisfied that reviewer comments have been addressed fully.

(Remarks on code availability)
Code runs appropriately and is very well documented for general use.

Open Access This Peer Review File is licensed under a Creative Commons Attribution 4.0 International License, which permits use, sharing, adaptation, distribution and reproduction in any medium or format, as long as you give appropriate credit to the original author(s) and the source, provide a link to the Creative Commons license, and indicate if changes were made.
In cases where reviewers are anonymous, credit should be given to 'Anonymous Referee' and the source.
The images or other third party material in this Peer Review File are included in the article's Creative Commons license, unless indicated otherwise in a credit line to the material. If material is not included in the article's Creative Commons license and your intended use is not permitted by statutory regulation or exceeds the permitted use, you will need to obtain permission directly from the copyright holder.
To view a copy of this license, visit https://creativecommons.org/licenses/by/4.0/

University of Bergen
Department of Biological Sciences
Faculty of Mathematics and Natural Sciences

Bergen, 26.02.2026

Subject: Revision of manuscript

Contact
Lotta Schultz
lotta.schultz@uib.no

Affiliation
Department of Biological Sciences
University of Bergen

Address
Thormøhlensgate 53A
Bergen, Norway

Dear reviewers,

Thank you for taking the time to review our manuscript and for providing such constructive and encouraging feedback. We greatly appreciate the careful attention you gave to the work and the suggestions and positive input you provided.

All your comments have been carefully considered and addressed, and the manuscript has been revised accordingly. We believe these changes have further strengthened the paper, so we are grateful for your careful revision of our work.

Major changes include:

1. Strengthening the main messages of the study. We restructured the Results section and revised the text to more clearly describe the key findings and their relevance (as suggested by Reviewer 3). In particular, we revised the sections *Data limitations* and *Future directions* to provide clearer take-home messages for the reader and to more explicitly highlight the broader relevance of the results for alpine biodiversity research.
2. Expanding the interpretations of how mountain biogeographic histories influence present-day patterns of alpine biodiversity (section *Perspectives on global alpine biodiversity*)
3. Replacing the term *alpine generalist* with *all alpine species* to improve conceptual clarity throughout the manuscript

Our detailed point-by-point responses are provided in blue under each comment below, and all changes are marked with track changes in the manuscript.

We hope that the revised manuscript now meets your and the editors' expectations and is suitable for publication in the journal.

Yours sincerely,
on behalf of all authors

Lotta Schultz

Reviewer #1 (Remarks to the Author):

Rev 1: This is a very impressive study, and very valuable global product from the GMBA Mountain Portal and other databases. Detailed empirical studies of significant but fragmented systems - such as the alpine biome - across the globe are scarce. This paper has a strength of being truly globally representative, and creates a baseline for additional studies and understanding on alpine areas globally. It speaks to global conventions and policy, with mountain-specific application for national/transboundary interest and action.

Dear reviewer,

Thank you for your time and positive review of our manuscript! We greatly appreciate your thoughtful feedback and comments. We have carefully incorporated your suggestions and we see the valuable improvements we could make thanks to your input. Below, we provide detailed responses to each of your comments:

Abstract
Rev 1: 0: I would add to the last sentence "suggesting diverse historical biogeographies of alpine areas". Something the result show is that alpine areas differ tremendously between them in terms of richness, as well richness between phyla. Area explains some it, but as aluded to, historical biogeography plays a tremendous role in explaining this.

Response: Thank you for this suggestion and we agree that historical biogeography plays a crucial role in shaping the patterns we find in our study. In the first version of the manuscript, we had been deliberately careful in not expanding into the likely causes of the observed patterns (as we do not present a study of causation and attribution on alpine biodiversity), but we certainly see the benefit of exploring potential causes to enrich the overall story. Therefore, we have revised the wording accordingly in line 96-98 as follows and additionally expanded in the section "Perspectives on alpine biodiversity" historical biogeography and the link to present day alpine biodiversity.

Revised sentence in the Abstract:

"Unlike the classical latitudinal gradient of biodiversity, alpine richness patterns show no consistent relationship with latitude, highlighting the importance of regional history, landscape structure, and biogeographical processes."

Main Text
Rev 1: 1. p.3, l.59 - change "thrive" to "occur".

Response: Revised as suggested. We slightly restructured the sentence in line 123-125 as follows:

"This information is crucial for identifying which species are true alpine specialists (i.e. restricted to alpine ecosystems) and which also occupy a wider range of elevations, including areas below the alpine zone[1,2]."

Rev 1: 2. p.8-9, ll.236-245 - important comments on historical biogeographies. The paper does lend itself to more interpretation perhaps on this. (Miocene)Pliocene-Pleistocene species pumps from climatic oscillations, uplift events, etc., and the data lends itself to potentially powerful global theory of island biogeography application. The background extinction rate in alpine areas compared to global change extinction rate/projected would add a powerful impetus to the paper's impact.

Response: Thank you for this important comment. As already stated in the previous response, we fully agree that linking alpine biodiversity patterns to biogeographic histories provides valuable context to enrich the findings of our paper. We have therefore expanded our interpretations of these patterns in the section "Perspectives on alpine biodiversity" (lines 276-308). However, adding additional analyses to

relate patterns to underlying processes would extend beyond the scope of the present paper, which is focused on compiling a new dataset and revealing current-day global alpine biodiversity. We see this work as the foundation that will enable such historical analyses in follow-up studies (we are currently in the process of doing so).

Revised paragraph:

"Perspectives on global alpine biodiversity

Our results reveal global variation in patterns of alpine biodiversity, characterised by distinct 'hotspots' and 'coldspots', the absence of a consistent latitudinal pattern, and differences in alpine specialisation among taxonomic groups. This raises key questions about the processes that shape alpine biodiversity. Many of these processes are likely linked to differences in geological history[3], mountain topography[4] and the legacy of past climate dynamics[5]. Repeated glacial advances and retreats reshaped alpine ecosystems through ice cover and erosion, altering their degree of connectivity and isolation over the last millions of years[5,6]. These dynamics interacted with mountain shape and topography to determine whether glacial and interglacial periods fragmented alpine ecosystems into isolated refugia or, conversely, expanded and connected alpine areas[4–6]. Such processes influenced species persistence, migration, hybridization, extinction, and speciation over evolutionary timespans, ultimately shaping patterns in alpine biodiversity[7].

Yet, important differences in these biogeographic processes exist among mountains. During glacial periods, extensive glaciers in the European Alps restricted alpine ecosystems to peripheral refugia around the border of the icesheet[8]. In contrast, alpine ecosystem connectivity increased in the Northern Andes[9] and African mountains[10] during these periods, although to varying degrees[11] shaped by mountain specific topography[4]. Mountain ranges with limited topographic relief, such as the Drakensberg, likely offered fewer stable refugia and reduced habitat heterogeneity, restricting the persistence of alpine communities through past climatic fluctuations[10], which may explain the lower absolute species richness observed in this region. In contrast, geologically young and topographically complex mountain ranges such as the Northern Andes, with their many peaks, ridges and high-elevation plateaus, created dynamic mosaics of high connectivity and fragmentation (sensu 'flickering connectivity system'[7]), providing refugia and cradles for species to persist and speciate[9,12,13]. These processes likely underlie the exceptionally high alpine biodiversity observed in these regions. A comparable pattern is evident in North America, where the western Cordillera had non-glaciated refugia during the Pleistocene, allowing species to persist and diversify, while the fully ice-covered eastern ranges lacked such refugia, leading to lower present-day alpine species richness with less alpine specialists[14].

Despite these hypothesized relationships between mountain biogeographic history and alpine biodiversity, the extent to which regional differences in mountain uplift and climate-driven range dynamics have shaped present-day alpine biodiversity (Fig. 2) remains largely unexplored in a quantitative framework."

Rev 1: 3. p.10, ll.280-282 - speaks to 2. above as well. e.g. Lower plant richness in the Drakensberg for instance is likely due to the alpine area only being "lower alpine", with an elevation depth of only c.400 m, due to the ancient, planed Gondwana land-surface on the summit (low summit relief, and limited topographic heterogeneity), probably resulting in a higher background extinction rate in an area with high climatic variability and alpine specialists needing to re-evolve post warm events.

Response: Thank you for this interesting suggestion. We agree and have changed the sentence following your suggestion in lines 293-296:

“Mountain ranges with limited topographic relief, such as the Drakensberg, likely offered fewer stable refugia and reduced habitat heterogeneity, restricting the persistence of alpine communities through past climatic fluctuations[10], which may explain the lower absolute species richness observed in this region.”

Rev 1: 4. p.10, "data limitations" etc. - personally I feel this section could be more punchy. The paper lays a good foundation, but next steps should be more clear - opportunities should be clearly outlined, and the paper could position itself as a "communication" more strongly for better support and action (data collection, policy impact, etc.). Make the strengths and weakness of the paper more succinct (less rambling), and outline what "could be" building on this data.

Response: Thank you for pointing out that this section needs more clarity. We agree with your suggestion and have revised the text accordingly in the sections “Data limitations” (line 309-336) and “Future directions” (line 337-361)

Revised paragraphs:

Data limitations

Our dataset lays the groundwork for future studies on alpine biogeography and future biodiversity assessment across the globe. However, like most large-scale biodiversity compilations, it holds certain limitations and biases. One is that the range maps we used rely on sampling efforts, which remain uneven across the globe and are often concentrated in regions with more established research infrastructure (e.g. USA, Australia, and Europe)[15]. Range maps are therefore more accurate in some regions than in others. In addition, the spatial resolution of these maps does not allow precise approximations of where species actually occur within individual mountain ranges or across elevational gradients.

To address these limitations, we incorporated elevational ranges as an additional dimension of species distributions, allowing us to identify which species are likely to be found in the alpine zone of each mountain range. A key strength of this approach lies in the involvement of regional experts who validated the regional elevational distribution and occurrence of species. At the time of analysis, 57% of the species lists were reviewed, reflecting the availability of experts across regions at the time of analysis (Supplementary Information Fig. 2). Nevertheless, the absence of validation in some regions may have left some inaccuracies in species elevational ranges and occurrences. For example, the comparatively high reptile richness observed in western North American mountain ranges, contrasted with the apparent absence of reptiles in eastern North American mountain ranges is likely an artefact, stemming from imprecise range maps and the lack of validation. Similarly, the global coverage for plants remains incomplete, with certain regions, such as Central Asia, still underrepresented (Supplementary Information Table 2), and with additional plant species likely remaining to be discovered, particularly in tropical mountain regions.

For birds, we used resident ranges rather than breeding ranges, a necessary compromise due to limited availability of high-resolution seasonal data. While this approach ensures broader global coverage and aligns with our aim to capture species that occupy alpine zones year-round, it excludes migrants that breed in alpine ecosystems but overwinter elsewhere, particularly in temperate regions.

Future directions

Our study presents a global synthesis of multi-taxon richness patterns of the alpine biome, providing new insights into the distribution and diversity of alpine vertebrates and plants. By integrating data across regions and taxa, we uncover a previously unrecognized global mosaic of alpine biodiversity, revealing distinct ‘hotspots’ and ‘coldspots’ of high-elevation biodiversity across taxonomic groups.

Our findings underscore the need to disentangle the regional dynamics of individual mountain ranges to understand how alpine biotas are shaped and reshaped by environmental change. Future research must move beyond broad approaches to probe the ecological processes underpinning alpine diversity,

including how diversification, dispersal barriers, and biogeographic legacies interact to define species pools, elevational limits, and their sensitivity to environmental changes.

Our open access dataset provides a foundation for advancing such future research. By providing comparable data across mountain systems worldwide, we aim to establish a consistent global baseline that can continue to be updated and spatially assessed in relation to climate projections, land-use scenarios and species' threat assessments along elevational gradients. Our work enables the identification of alpine ecosystems where biodiversity is concentrated, underrepresented or potentially at risk, to ultimately support the development of region-specific conservation priorities and management actions.

Finally, we see this work as a catalyst for continued collaboration with regional experts to improve data coverage, refine elevational ranges, and expand taxonomic representation and accuracy. Only by connecting regional expertise worldwide, we can build the evidence base needed to inform global policy frameworks, such as the IPCC, IPBES, and the Kunming–Montreal Global Biodiversity Framework, and ultimately safeguard high-elevation ecosystems and their contributions to future generations.

Methods:

Rev 1: 1. Were species extinct in historical/near-historical times included? This can affect some results, e.g. perhaps in New Zealand (Moas, other land birds)? Extirpated species from some alpine areas but still present in others e.g. Lammergeier? This may change some data results and also emphasize human impact on alpine species (sensu Global Change not just climate change).

Response: Thank you for these interesting questions. We agree that our methods were not clear on this point. We did not include any extinct or historically extirpated species in our dataset; only extant species, as defined by BirdLife International, were considered. We have clarified this now more explicitly in lines 402 and 404. We agree that including extinct or regionally extirpated species could provide important insights into human impacts on alpine biodiversity. However, assembling such data consistently across all taxonomic groups and regions would be very challenging at the global scale. We therefore consider this an important idea for follow-up research.

Revised paragraph:

"For mammals and reptiles, we gathered global native distribution ranges for all extant species from the Mammal Diversity Database (MDD; n = 6,544 spp.)[16], and the Global Assessment of Reptile Distribution (GARD, version 1.7; n = 10,064 spp.)[17,18], respectively. For all extant birds, we used the BirdLife International native resident distribution ranges defined as the area where species are known or thought to be resident throughout the year or visit regularly during the breeding season (n = 11,195 spp.)[19]."

Rev 1: 2. I would suggest corroborating the delimitation of alpine areas used in the methods with a climatic one, e.g. 6oC isotherm. This would give the delimitations more weight and justification, and or suggest modifications.

Response: Thank you for this very relevant observation. The delineation of alpine areas we use in our study is, as you suggest, indeed already a climatic one. We applied the Köppen–Geiger climate maps, specifically Class 29 (ET; Polar, Tundra) that defines areas where mean warmest-month temperatures remain below 10 °C. The ~6 °C treeline isotherm you mention is instead often used as the physiological threshold for the survival of individual trees. Rather than the treeline (where individual trees could potentially still grow), our approach is based on the upper forest line (i.e., the climatic limit of continuous closed montane forest), and this boundary is better captured by the 10 °C threshold [2]. Importantly, we fully agree with you that using the climatic limit of tree growth is essential for an ecological definition of the alpine biome, and we believe our approach already reflects this. To make our delineation clearer, we have added the following sentence to the methods (line 376).

“This class corresponds to areas where the mean temperature of the warmest month is below 10 °C, i.e. conditions that physiologically limit tree growth.”

We also revised and strengthened our rational for using the upper forest line in line 378-3854:

“In this context, we refer to the outer boundary of these polygons as the upper forest line (UFL), rather than the treeline. The latter includes the uppermost tree-size individuals, whose growth is strongly influenced by local site conditions[20]*. Modelling and predicting its precise location can therefore be challenging due to patchiness and limited spatial resolution in global climate datasets. In contrast, using the upper limit of the continuous montane forest provides a clearer ecological boundary between forest and alpine ecosystems and a more consistent representation at the scale of our climate and distribution data, making it more appropriate for our approach.”*

Rev 1: 3. The latitudinal question is NB, and well-integrated into the study. However perhaps it should be mentioned that although climate is "similar" across alpine areas (e.g. mean ann temp 6oC), latitude plays a large role in local abiotic includ. climate processes due to daylight length, and daily temp fluctuations vs seasonal temp fluctuations. Tropical alpines have "summer every day and winter every night" with little insolation variability, while temperate and polar alpines have extended seasonal insolation differences driving highly variable daylight lengths and seasonal temperature differences.

Response: Thank you for bringing up this important and interesting point. The role of local abiotic processes in shaping latitudinal gradients is indeed something we had missed explaining. We have now clarified this in the text in line 258-262.

Revised paragraph:

“If climate alone would drive latitudinal patterns of biodiversity, the relationship between species richness and latitude should weaken. If, however, other latitude-linked factors, such as differences in diurnal and seasonal variations in temperature[21,22]*, also shape biodiversity, a latitudinal gradient in alpine species richness may still emerge.”*

Rev 1: 4. Re. birds, long-distance migrants are mentioned. Local and regional migrants are worth a mention, e.g. alpine breeding endemics that disperse seasonally to other areas within-continent, or to lower elevations nearby.

Response: Thank you, we agree with your suggestion and include it in line 407-413.

Revised paragraph:

“Due to limited data availability, we focused on resident distribution ranges as defined by BirdLife International, which represent the year-round geographic ranges of non-migratory species and include their breeding areas[19]*. Long-distance and local breeding migrants that disperse seasonally were excluded because our aim was to include only those species that use the alpine zones as their primary habitat throughout the year. A valuable complementary reference to our data is the list of alpine breeding birds available from DeZwaan et al. (2020), which focuses exclusively on breeding occurrences*[23]*.“*

Figures etc.
Rev 1: 1. Extended Data Table 3 - standardise use of capitals and spacing in column headings.

Response: Changed. Extended Data Table 3 has been standardized.
Rev 1: 2. Other figures and diagrams suitable etc.

We expect that the reviewer left a comment pending here to complete. Otherwise, we hope that our figures and diagrams are suitable for publication and we would be happy to make any adjustments needed in the

meantime.

Reviewer #2 (Remarks to the Author):

I co-reviewed this manuscript with one of the reviewers who provided the listed reports. This is part of the Nature Communications initiative to facilitate training in peer review and to provide appropriate recognition for Early Career Researchers who co-review manuscripts.

Dear reviewer,

Thank you for taking the time to review our manuscript and code. We greatly appreciate your positive feedback and are very supportive of the co-reviewing program for Early Career Researchers. This is an excellent initiative to provide recognition and training in peer review!

Reviewer #2 (Remarks on code availability):
Rev 2: The code was tested and it runs very well. The instructions are adequate and easy to follow. All required packages installed without any challenges.

Response: Thank you for taking the time to review our R Code in detail. We have spent substantial amount of time making it user friendly and overall reproducible, so we are happy to hear your positive feedback.

Reviewer #3 (Remarks to the Author):

STRENGTHS:

Rev 3: This study seeks to enhance our comparative understanding of alpine diversity by providing a "comprehensive, expert-validated dataset" on global alpine biodiversity for several, broad taxonomic groups (vascular plants, mammals, birds, and reptiles). There is absolutely a need for more data in this field and the authors are providing critical information by helping to fulfill that need. One of the main strengths of a dataset such as this is the "expert-validation" aspect, which is often missing from these types of big-data analyses. Another strength is that the authors parse species into alpine generalist and specialist categories, which has been noted in the literature as a method to obtain more biologically-relevant categories.

The authors' results corroborate and build off previous literature examining broad spatial biodiversity patterns among alpine species. They find that many alpine species are "super-specialists," often confined to a single mountain range, which underscores the authors' assertions regarding the pivotal ecological role these ecosystems play and their importance for consideration in conservation planning.

Consistent with previous research, the authors find that Neotropical alpine biomes are biodiversity hotspots with a high degree of specialists and higher latitude alpine regions are less biodiverse with proportionally more generalist species. However, they also astutely illustrate and explain the decoupling of alpine biodiversity patterns from any traditional latitudinal gradient, offering a nice comparison of latitudinal patterns when climatic factors are more uniform. This enhances our overall understanding of alpine biodiversity as well as the underlying mechanisms that drive the traditional latitudinal diversity gradient. The authors are sensitive to the historical factors that likely have driven these overall patterns (phylogenetic as well as climatic and geologic).

Dear Reviewer,

We are very thankful for the feedback you have given on our manuscript. We appreciate that you took the time to read everything thoroughly and highlighted important points. Your suggestions were very valuable and helped improve the manuscript substantially. Please find our responses to your comments below.

WEAKNESSES/ CONSIDERATIONS:

Rev 3: I think there are critical science philosophy questions to consider about whether we, as researchers, really should expect one study to "do it all" in this manner and be able to provide a comprehensive dataset for such disparate groups – or even whether such data are comparable across these groups – however that is not a criticism of the authors approach or goals, but a reflection of the overall culture in this field. Nevertheless, I caution against calling this (or any) dataset "comprehensive." No matter how extensive or carefully curated, there will always be gaps. The authors do a good job of explaining the gaps in their own dataset, even as they call it comprehensive, for example citing the artefacts in their reptile data. Additionally, as research continues, current datasets always give way to new discoveries and refined methodologies, making old datasets obsolete very rapidly. The authors have curated a very refined and thoughtful comparative dataset, and I think it suffices for them to refer to it as such. Calling it "comprehensive" may invite criticisms from other scientists where there did not need to be any.

Response: We understand the point raised by the reviewer. We didn't mean to imply that our dataset is complete or free of errors. Following your suggestion, we removed the word "comprehensive" throughout the manuscript. In the Abstract we replaced "comprehensive" with "curated" (line 91).

Furthermore, we also wish to emphasize that we see this dataset as a resource developed through ongoing collaboration with regional experts, with the aim of improving data accuracy and coverage.

Rev 3: Overall, this study has contributed greatly and built a very nice dataset that is solid and usable by the community at large. However, although I truly enjoy this paper and its presentation, I am still left wondering “what’s the main takeaway”? What is the big picture message I should have now that I didn’t have before and what has the community learned from this that it did not already know?
The biggest finding seems to be the latitudinal patterns of biodiversity (or lack thereof). At the same time, though, the authors main framing in the abstract and introduction seems to highlight the conservation and biodiversity preservation aspect. I would like to see the authors tie their results to their big picture message more and give the readers a clear take-home and assessment of the import of the results overall for our understanding of alpine biodiversity. Right now, the main message seems to be, essentially, that there is a lot of heterogeneity, however we already know that is the norm for biological communities. So, what should we be doing with this heterogeneity? How do we apply that knowledge when thinking about conservation and safeguarding alpine ecosystems?

Response: Thank you for this thoughtful comment and after close consideration we agree that our main messages could have been made much stronger. Therefore, we revised the abstract as well as the structure and clarity of our main paragraphs describing the results: “Global hotspots and coldspots of alpine biodiversity”, “Complex patterns across mountains and taxonomic groups”, “Latitudinal trends of richness”, “Perspectives on global alpine biodiversity”, as well as paragraphs “Limitations” and “Future direction”. We aimed to clarify the take-home messages and strengthen the discussion of the implications of our results.

METHODS:
Rev 3: L363: Regarding a UFL vs treeline approach, was there any attempt made to see if the UFL approach outlined here produced very different results from a treeline approach? This could be done with just those areas for which the treeline approach can be implemented relatively easily as a validation check. If the two methods do not produce substantially different results, I think that is worth knowing/ stating for the future as the authors’ UFL approach is computationally much easier.

Response: Thank you for this important question. We did not make a direct comparison between the UFL and treeline approaches. However, during the process of the development of this manuscript, we have had in-depth discussions within our author team about which ecological boundary and naming would be most appropriate for our study. In the end, we opted for the UFL because it marks the climatic limit of continuous montane forest and therefore provides a clearer ecological boundary between forest and alpine ecosystems. The treeline instead reflects the uppermost scattered individual trees, which are more strongly influenced by local conditions and thus represent a less consistent ecotone. In addition, we know of examples of tree species (e.g. Podocarpus in the Andes) that are far within the elevational range of the alpine biome and consequently using the treeline elevation would substantially reduce the estimated area for the alpine biome. For our purpose of broadly categorizing alpine species on the global scale, we therefore considered the UFL to be the more ecologically meaningful “threshold”. We recognize that our ecological reasoning for opting for the UFL approach was not clear in our methods and we therefore revised the following paragraph in line 373-385

Revised paragraph:

“To spatially delineate the alpine biome globally, we followed the climate-delimitated approach by Kandziora et al. (2024)[24] based on the high-resolution Köppen-Geiger climate maps[25]. Specifically, we extracted Köppen-Geiger Class 29 (ET; Polar, Tundra) for each GMBA mountain range to represent the alpine biome. This class corresponds to areas where the mean temperature of the warmest month is below 10 °C, i.e. conditions that physiologically limit tree growth. In this context, we refer to the outer boundary of these polygons as the upper forest line (UFL), rather than the treeline. The latter includes the uppermost tree-size individuals, whose growth is strongly influenced by local site conditions[20]. Modelling and predicting its precise location can therefore be challenging due to patchiness and limited spatial resolution in global climate datasets. In contrast, using the upper limit of the continuous montane forest

provides a clearer ecological boundary between forest and alpine ecosystems and a more consistent representation at the scale of our climate and distribution data, making it more appropriate for our approach."

Rev 3: L438: This is probably the single greatest strength of this dataset, and I think the authors have done a really great job of pulling this off for such a wide variety of broad taxonomic groups.

Response: Thank you for this kind comment. It has indeed been a massive effort, and we appreciate the reviewer's positive statement.

Rev 3: L473: I agree with the authors here that this approach, though necessary, should be interpreted with caution. Particularly in cases where important inferences are being made solely based on this approach.

Response: Thank you for agreeing with this section. We have been careful to frame our interpretations with caution and refer to the section "Estimating alpine vertebrate richness in the alpine biome worldwide" in the methods (lines 485-492) where we discuss the limitations of the SAR approach.

Rev 3: L493: It is unclear to me from this description and Fig. 1 if experts also validated the vascular plant portion of the dataset. If not, my question would be why not? Was it not needed due to the quality of the data or was it that experts were not sought out.

Response: Thank you for this comment. The plant data were not re-validated by our regional experts because they were already compiled as expert-based regional checklists in a previous effort. These datasets underwent quality control by our key collaborators leading the GIFT database initiative before they were integrated into the database. We therefore relied on this existing validation rather than duplicating the process. For clarity, we revised the sentence in lines 517 and 526-528 of our methods section.

Revised paragraph:

"We first gathered regional plant checklists assembled by experts for a previous study[22] and added them to the Global Inventory of Floras and Traits (GIFT) database[26]. Using the GIFT R package[27], we then sourced 80 regional checklists of native vascular plants from GIFT version 3.2 for mountains that included alpine biome and contained regional-specific elevational data (Supplementary Information Table 3). GIFT checklists are taxonomically standardized using the World Checklist of Vascular Plants (WCVP) taxonomic backbone[28] and cover various geographic and political units. GIFT is continually updated with new regional checklists. However, at the time of our analysis, plant data was unavailable for some mountain ranges (e.g. in Central Asia) for which vertebrate data was available, leading to occasional mismatches in data coverage between plants and vertebrates in our results. Unlike our vertebrate data, the plant checklists were not revalidated for this study as they had already undergone quality check before their integration into the GIFT database."

Rev 3: L557: For panels C/D, I find the gray shading hard to follow. That might be intentional since no strong pattern is observed, but I would prefer a different color scheme.

Response: We changed the color scheme in panels Fig 2. C/D to the following:

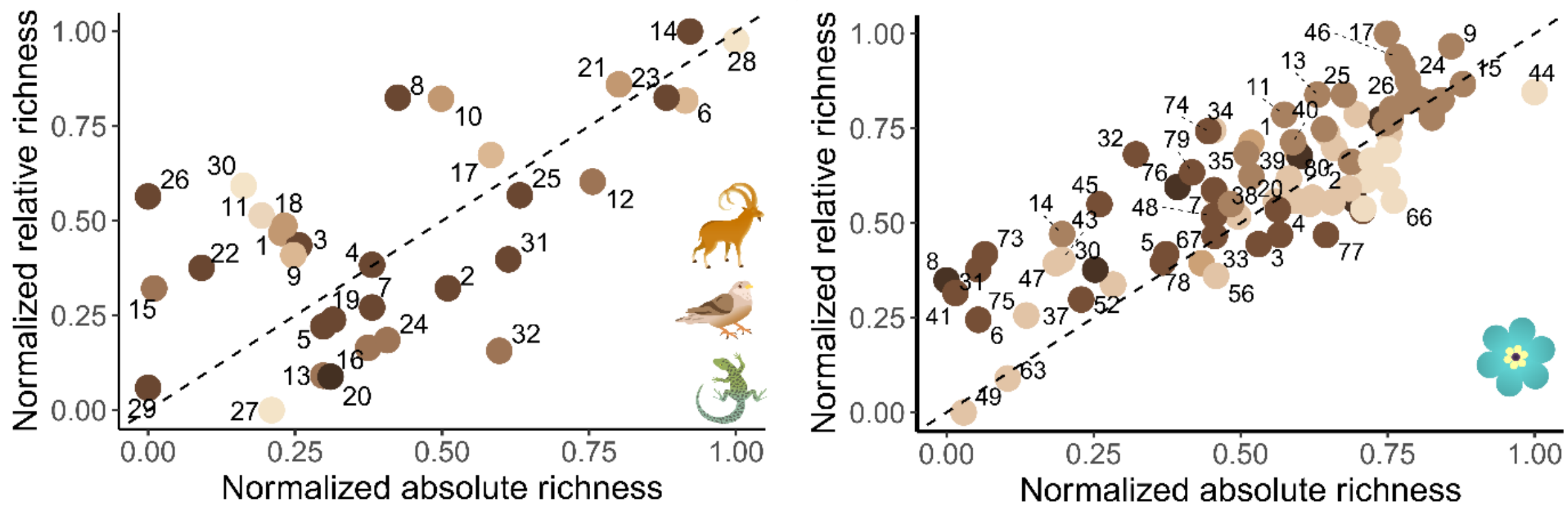

Reviewer #3 (Remarks on code availability):

Rev 3: I reviewed the GitHub broadly, not necessarily line by line in each file of code. However, overall, I think this is an excellent repository and resource for the community. It is well organized and documented, appears to follow best practices, and there are tutorials and other training resources to allow users of all experience levels to use the provided code.

Response: Thank you for reviewing our GitHub. We paid special attention to having it well organized and following best practices so we are happy to see that this effort paid off. Thanks!

References:

1. Testolin, R. *et al.* Global patterns and drivers of alpine plant species richness. *Glob. Ecol. Biogeogr.* **30**, 1218–1231 (2021).
2. Körner, C., Paulsen, J. & Spehn, E. M. A definition of mountains and their bioclimatic belts for global comparisons of biodiversity data. *Alp. Bot.* **121**, 73–78 (2011).
3. Ding, W. *et al.* The asynchronous rise of Northern Hemisphere alpine floras reveals general responses of biotic assembly to orogeny and climate change. *Sci. Adv.* **11**, eadz1888 (2025).
4. Elsen, P. R. & Tingley, M. W. Global mountain topography and the fate of montane species under climate change. *Nat. Clim. Change* **5**, 772–776 (2015).
5. Flantua, S. G. A. *et al.* Snapshot isolation and isolation history challenge the analogy between mountains and islands used to understand endemism. *Glob. Ecol. Biogeogr.* **29**, 1651–1673 (2020).
6. Steinbauer, M. J. *et al.* Topography-driven isolation, speciation and a global increase of endemism with elevation. *Glob. Ecol. Biogeogr.* **25**, 1097–1107 (2016).
7. Flantua, S. G. A. & Hooghiemstra, H. Historical Connectivity and Mountain Biodiversity. in *Mountains, Climate and Biodiversity (eds C. Hoorn, A. Perrigo & A. Antonelli)* 171–185 (2018).
8. Schönswetter, P., Stehlik, I., Holderegger, R. & Tribsch, A. Molecular evidence for glacial refugia of mountain plants in the European Alps. *Mol. Ecol.* **14**, 3547–3555 (2005).
9. Flantua, S. G. A., O'Dea, A., Onstein, R. E., Giraldo, C. & Hooghiemstra, H. The flickering connectivity system of the north Andean páramos. *J. Biogeogr.* **46**, 1808–1825 (2019).
10. Brochmann, C. *et al.* History and evolution of the afroalpine flora: in the footsteps of Olov Hedberg. *Alp. Bot.* **132**, 65–87 (2022).
11. Sklenář, P., Hedberg, I. & Cleef, A. M. Island biogeography of tropical alpine floras. *J. Biogeogr.* **41**, 287–297 (2014).
12. Rahbek, C. *et al.* Building mountain biodiversity: Geological and evolutionary processes. *Science* **365**, 1114–1119 (2019).

13. Antonelli, A. *et al.* Geological and climatic influences on mountain biodiversity. *Nat. Geosci.* **11**, 718–725 (2018).

14. Brunsfeld, S., Sullivan, J., Soltis, D. E. & Soltis, P. S. Comparative Phylogeography of Northwestern North America: a Synthesis. *Spec. Publ.-Br. Ecol. Soc. 14 319-340* **14**, (2001).

15. Hughes, A. C., Orr, M. C., Yang, Q. & Qiao, H. Effectively and accurately mapping global biodiversity patterns for different regions and taxa. *Glob. Ecol. Biogeogr.* **30**, 1375–1388 (2021).

16. Marsh, C. J. *et al.* Expert range maps of global mammal distributions harmonised to three taxonomic authorities. *J. Biogeogr.* **49**, 979–992 (2022).

17. Caetano, G. H. D. O. *et al.* Automated assessment reveals that the extinction risk of reptiles is widely underestimated across space and phylogeny. *PLOS Biol.* **20**, e3001544 (2022).

18. Roll, U. *et al.* The global distribution of tetrapods reveals a need for targeted reptile conservation. *Nat. Ecol. Evol.* **1**, 1677–1682 (2017).

19. BirdLife International. BirdLife International and Handbook of the Birds of the World (2022) Bird species distribution maps of the world. Version 2022.2. Available at http://datazone.birdlife.org/species/requestdis. (2022).

20. Körner, C. The alpine life zone. in *Alpine Plant Life: Functional Plant Ecology of High Mountain Ecosystems* 9–20 (Springer, Berlin, Heidelberg, 2003). doi:10.1007/978-3-642-18970-8_2.

21. Stevens, G. C. The Latitudinal Gradient in Geographical Range: How so Many Species Coexist in the Tropics. *Am. Nat.* **133**, 240–256 (1989).

22. Gallou, A. *et al.* Diurnal temperature range as a key predictor of plants' elevation ranges globally. *Nat. Commun.* **14**, 7890 (2023).

23. de Zwaan, D. R. *et al.* GABB: A global dataset of alpine breeding birds and their ecological traits. *Sci. Data* **9**, 627 (2022).

24. Kandziora, M. *et al.* The ghost of past climate acting on present-day plant diversity: Lessons from a climate-based delimitation of the tropical alpine ecosystem. *J. Syst. Evol.* **62**, 275–290 (2024).
25. Beck, H. E. *et al.* High-resolution (1 km) Köppen-Geiger maps for 1901–2099 based on constrained CMIP6 projections. *Sci. Data* **10**, 724 (2023).
26. Weigelt, P., König, C. & Kreft, H. GIFT – A Global Inventory of Floras and Traits for macroecology and biogeography. *J. Biogeogr.* **47**, 16–43 (2020).
27. Denelle, P., Weigelt, P. & Kreft, H. GIFT—An R package to access the Global Inventory of Floras and Traits. *Methods Ecol. Evol.* **14**, 2738–2748 (2023).
28. Govaerts, R., Nic Lughadha, E., Black, N., Turner, R. & Paton, A. The World Checklist of Vascular Plants, a continuously updated resource for exploring global plant diversity. *Sci. Data* **8**, 215 (2021).
